# The construction of a hypoxia-based signature identified CA12 as a risk gene affecting uveal melanoma cell malignant phenotypes and immune checkpoint expression

**DOI:** 10.3389/fonc.2022.1008770

**Published:** 2022-09-26

**Authors:** Yan Yin, Wei Du, Fei Li

**Affiliations:** ^1^ Department of Ophthalmology, The Second Affiliated Hospital of Shandong First Medical University, Taian, China; ^2^ Department of Ophthalmology, The Shandong Second Rehabilitation Hospital, Taian, China; ^3^ Department of Medicine, Shandong First Medical University, Taian, China

**Keywords:** uveal melanoma, hypoxia, immune, carbonic anhydrase 12, risk score

## Abstract

Uveal melanoma (UM) is a deadly intraocular neoplasm in the adult population and harbors limited therapeutic effects from the current treatment. Here, we aimed to investigate the role of hypoxia in UM progress. We adopted the Cancer Genome Atlas data set as a training cohort and Gene Expression Omnibus data sets as validating cohorts. We first used consensus clustering to identify hypoxia-related subtypes, and the C1 subtype predicted an unfavorable prognosis and exhibited high infiltration of immunocytes and globally elevated immune checkpoint expression. Besides this, the patients with the C1 subtype were predicted to respond to the PD-1 treatment. By the least absolute shrinkage and selection operator algorithm, we constructed a hypoxia risk score based on the hypoxia genes and identified 10 genes. The risk score predicted patient survival with high performance, and the high-risk group also harbored high immunocyte infiltration and immune checkpoint expression. Furthermore, we confirmed that the risk genes were upregulated under hypoxia, and knockdown of CA12 inhibited the epithelial–mesenchymal transition process, clone formation ability, and G1/S phase transformation of the UM cells. The CD276 was also downregulated when CA12 knockdown was performed. These results validate the prognostic role of the hypoxia signature in UM and demonstrate that CA12 is a critical factor for UM cell progression as well as a target to improve immunotherapeutic effects. We believe our study contributes to the understanding of hypoxia’s roles in UM and provides a novel target that will benefit future therapeutic strategy development.

## Introduction

Uveal melanoma (UM) is a rare tumor type among the population, but it is the most common intraocular neoplasm in the adult population with high malignancy. UM harbors a morbidity of approximately five cases per million per year, and 90% of UM originates from the choroid (1). Nearly 50% of UM metastases to the liver during tumor development, and early stage intervention, such as chemoembolization and surgical excision, controls tumor progression, but mostly prognoses of UM returns unfavorable due to the limited therapeutic strategy effects. More efficient approaches for improving the therapeutic effects or prognostic management are urgently required.

Molecular pathogenesis and targeted therapy have been novel research topics and promising strategies to prevent UM processes or improve patient survival (2). Currently, many molecular features have been applied to indicate the patient prognostic diversity, such as that active mutation of the Gα11/Q pathway drives the tumorigenesis of UM and *BAP1, SF3B1*, and *EIF1AX* mutant precited metastatic progression (3). Moreover, various studies are still carried on to discover novel molecular targets for UM (4–6). Many targeted therapy–based clinical trials have been conducted, whereas no mature approach has been proven for application, indicating the urgent need for more effective strategies for UM treatment (7).

Hypoxia, characterized by insufficient tissue oxygenation, is a critical risk factor in cancer, for its connection with various hallmarks of cancers, including angiogenesis, metabolism programming (8), and immunosuppression (9), contributing to the progression of cancer and poor prognosis. To overcome the hypoxia-related signaling in cancers, many hypoxia-targeted therapies were developed (10). In UM, hypoxia has been suggested to correlate with angiogenesis, invasion, and autophagy, indicating its significant role in UM. Besides this, many drugs were discovered to gain therapeutic effects, mostly based on HIF and angiogenesis (11). Interestingly, a study has demonstrated the effects of hypoxia stress on monocyte migration and characteristics (12); this implies the association between hypoxia and immunity of UM, whereas their intercorrelation is far from understood.

In this study, we used a hypoxia-based signature to identify novel hypoxia-based subtypes and build a prediction model for patient prognosis, and the biological involvement of hypoxia in UM was investigated by functional enrichment, and its correlation with immunocyte, immune checkpoints were explored. Moreover, we conducted a series of experiments to validate hypoxia’s effects on tumor cell phenotype and immune checkpoint expression. We hope this study will reveal a novel pathological mechanism of hypoxia in UM and provide alternative therapeutic targets for UM patient treatment.

## Materials and methods

### Data acquisition

The UM sample RNA expression and clinical information were obtained from the Cancer Genomes Atlas and were used as a training cohort, and UM samples from the Gene Expression Omnibus, GSE22138 and GSE84976, were downloaded as validating cohorts. The hypoxia, gene ontology (GO), and Kyoto Encyclopedia of Genes and Genomes (KEGG) gene sets were retrieved from the Gene Set Enrichment Analysis (GSEA) online database. The compounds used for potential drug identification were obtained from the GDSC database. The tumor immune dysfunction and exclusion (TIDE)-related calculation was performed on the TIDE online web tool.

### Consensus clustering of UM samples by the hypoxia gene sets

We used the hypoxia gene sets to cluster the UM training cohort by the “ConsensusCluster” R package with the best *k* value and visualized the results by a principal component analysis (PCA) plot. For the clusters obtained, we used survival analysis to evaluate their prognostic difference. In a heat map, an expression of the hypoxia genes in all samples divided by the clusters were also presented. Subsequently, we investigate the diversity of cancer hallmarks between the clusters using gene set variation analysis (13) and “Hallmark” gene sets downloaded from GSEA.

### Immune diversity between clusters

To investigate the immune diversity between the clusters of UM, we analyzed the 28 types of immunocyte infiltration of all samples using single sample GSEA (ssGSEA). We also compared the immune checkpoint expression differences between clusters. The results were presented in heat maps and box plots.

### Drug IC50 calculation and immunotherapy analyses

To identify novel drugs for hypoxia cluster–based targets, we downloaded the compound information, cell line expression matrix, and cell line testing results to predict the drug IC50 for all samples by the R package “pRRophetic” (14), and the IC50 values were compared between clusters in box plots. Besides this, we analyzed the immunotherapeutic effects of the samples by TIDE analyses, including the dysfunction, exclusion, IFNG, and TIDE score calculation conducted on the TIDE web tool. We also analyzed the correlation between PD-1, CTLA4 response, and clusters to explore the immunotherapeutic potential of the clusters.

### Biological diversity comparison between clusters

To compare the biological diversity between samples in different clusters, we first used the “limma” R package to filter the differentially expressed genes (DEGs) between clusters. Then, the DEGs were annotated according to the gene set annotation downloaded from GSEA, including KEGG and biological process (BP), cellular component (CC), and molecular function (MF) in GO.

### Hypoxia Least absolute shrinkage and selection operator (LASSO) risk score construction

The hypoxia gene sets were applied to the LASSO regression (15) to reduce the number of parameters and construct a prognostic model. The risk score of each sample was calculated as the sum of the coefficient multiplied by the expression of each gene. We divided the samples into high- and low-risk groups according to the median risk score of the cohort. The survival time and risk score gene expression were presented in order of the risk scores. We checked the prognostic value of the risk score by survival analysis and receiver operating characteristics curve (ROC) and, finally, built a nomogram integrating risk score, age, gender, and stage, and its prognostic value was estimated by ROC and calibration curve.

### The immune diversity between risk scores and potential drug identification

To discover the immune diversity between the risk groups, we used ssGSEA to analyze the immunocyte infiltration differences as well as the immune checkpoint expression variation (16), the results were presented by box plots. The correlation between CA12 and CD276 was quantified on GEPIA2.0, an online tool for cancer investigation. Besides this, we compared the Genomics of Drug Sensitivity in Cancer (GDSC) (17) compound IC50 values of the two groups, and the cMap was also utilized to filter potential drugs.

### Cell culture and siRNA transfection

The highly aggressive MUM2B cells were cultivated in DMEM with 10% FBS, maintained under 5% CO_2_ and 37°C and digested when they were 80% confluent. For the hypoxia culture, we cultivated the cells in the hypoxia incubator for 24 h. Then, the cells were planted into a 12-well plate and transfected with CA12 siRNAs and lip3000 transfection reagent. After 48 h transfection, the cells were harvested and counted for further experiments. The sequence of siRNA1 and siRNA2 was provided by Zhao et al. and Huang et al, respectively (18, 19).

### RT-qPCR detection of the mRNA levels

The cells were washed with PBS and lyzed in trizol for 10 min. The RNA was collected and extracted using chloroform. After centrifugation and supernatant collection, the RNA was precipitated with isopropanol, followed by sequential washing with 80% ethanol and absolute ethanol. When the RNA was dried naturally, a quantification by a microplate reader was then conducted. Subsequently, the genomic DNA was removed, and RNA reverse transcription was performed. Finally, the 10 μl mixed system per well containing 1μl cDNA, 0.4μl primers, 5μl SYBP, and 3.2μl RNA-free water was prepared, and the RT-qPCR was performed.

### Transwell migration assay

The harvested cells were resuspended with 200 μl FBS (1%) and planted into the upper chamber of the transwell with 1×10^4^ cells per well (Corning Incorporated, Corning, NY, USA). The lower chamber was filled with 500 μl DMEM with FBS (20%). After cultivation for 24 h, the upper chamber was slightly washed with PBS three times and fixed with crystal violet. After 30 min, the unmigrated cells were wiped off using a cotton swab and left to dry for microscopy.

### CCK8 assay

The harvested cells were resuspended using DMEM with FBS (10%) and seeded in a 96-well plate for the CCK8 assay. After cell adherence, the previous medium was replaced by DMEM with a 10% CCK8 reagent (GK3607-500T, GeneView, DingGuo Company, Changsha, China) containing no FBS. After 2 h of cultivation, the OD value was detected using the microplate reader. The absorbance was measured at 450 nm.

### Clone formation assay

As described, we used DMEM containing FBS (10%) to resuspend the cells and seeded them in the six-well plate, with 1000 cells per well. After 14 days of cultivation, the medium was removed and washed with PBS. Subsequently, the cells were fixed with crystal violet for 30 min.

### Flow cytometric analysis

The digested cells were collected in EP tubes. We centrifuged the cells to remove the medium and washed the cells with precooled PBS. Then, the cells were fixed using 70% ethanol under 4°C overnight. Subsequently, the ethanol was removed and washed by precooled PBS again and stained with propidium iodide (PI, 20X), RNase (50X), and staining buffer (C1052, Beyotim Biotechnology Co. Ltd,Nantong, China). The mixed system was incubated from light under 37°C for 30 min and detected by the flow cytometer. The data were analyzed by FlowJ software.

### Western blot detection

The cells were lysed using the RIPA lysis buffer and centrifuged to obtain the protein supernatants. The nuclear in the samples were then further lysed by ultrasound. After being mixed with loading buffer, the samples were loaded, and electrophoresis and membrane transformation were sequentially performed. The membrane was then blocked with skim milk powder (5%) and incubated under 4°C overnight with the primary antibodies (sources listed in Supplementary Table S1). The next day, the membrane was washed with TBST three times and incubated with the second antibodies for 1 h. Finally, the protein bands on the membrane were detected using the chemiluminescence detection system after TBST washing. The results were quantified by the ImageJ software.

### Statistical analyses

The bioinformatic analyses were performed on the R software. The Kaplan–Meier curve and log-rank test were used for survival analyses. Cox regression calculated the HR of each factor. ROC and the calibration curve estimated the predictive discrimination and calibration, respectively. Student’s *T*-test compared the expression differences between groups. Two-way ANOVA tested the CCK8 results. Spearman’s correlation coefficient was used to quantify expression correlation between genes. *P* <.05 was defined as statistically significant.

## Results

### Unsupervised clustering identified a cluster with a worse prognosis and upregulated hallmark features

**Figure 1:**
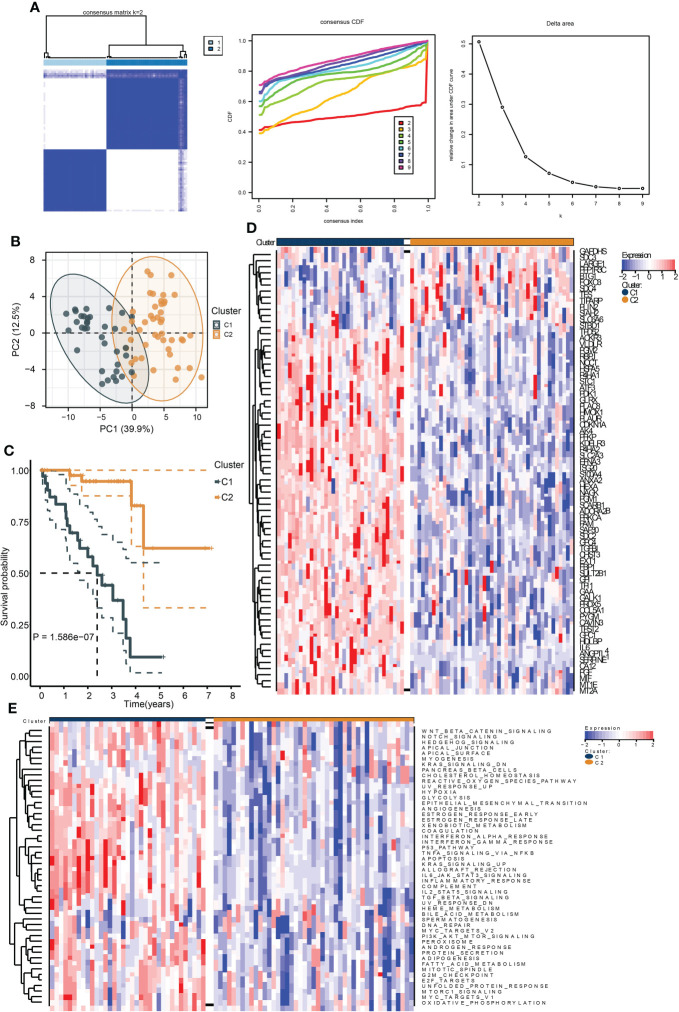
Identification of hypoxia-related subtypes of UM. **(A)** Consensus clustering heat map (left), CDF (middle), and the relative change in area under CDF curve (right) of the TCGA UM samples. **(B)** PCA plot showing the division results of consensus clustering. **(C)** Kaplan–Meier curve indicates the ability of subtypes to separate patient survival rate. **(D)** The expression heat map of the genes in hypoxia signature. **(E)** The enrichment heat map of the “hallmark” gene sets based on the DEGs between the subtypes.

To identify the hypoxia-associated clusters in UM. We used “ConsensusCluster” to conduct the unsupervised clustering of the training cohort. We selected the *k* value as 2, the samples were perfectly divided into two clusters (**Figure 1A**), and the PCA plot exhibited that the two clusters were separated into two groups (**Figure 1B**).

We then performed survival analysis to test the prognostic differences between the two clusters. As a result, cluster C1 exhibited a worse survival rate (**Figure 1C**). Moreover, most of the hypoxia-related genes were highly expressed in cluster C1 (**Figure 1D**). The ssGSEA results of the “Hallmark” gene sets of all samples showed that most of the cancer hallmark pathways were upregulated in cluster C1. Notably, many immune-related pathways were enriched in cluster C1, including IL6-JAK-STAT3, IL2-STAT5, TGF-β, interferon-α/γ-response, and TNFA-related signaling pathways (**Figure 1E**). These results indicated that hypoxia played a critical role in UM development, and these were associated with cancer immunity.

### Hypoxia-divided clusters presented a diverse immune status

**Figure 2:**
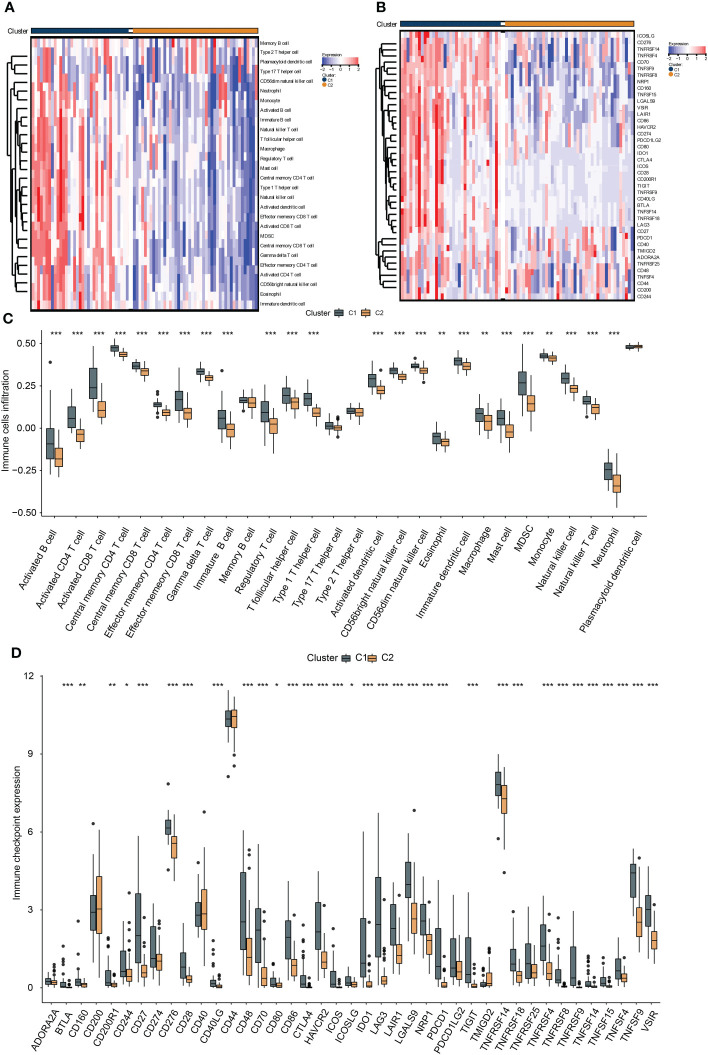
The association between hypoxia and immunocyte and immune checkpoints. The heat map presents the immunocyte infiltration difference **(A)** and the expression variation of the immune checkpoints **(B)** between subtypes, and their quantification results of immunocyte infiltration **(C)** and immune checkpoint expression **(D)** were exhibited in the box plots, *, **, *** represents p-value < 0.05, 0.01, and 0.001, respectively.

**Figure 3:**
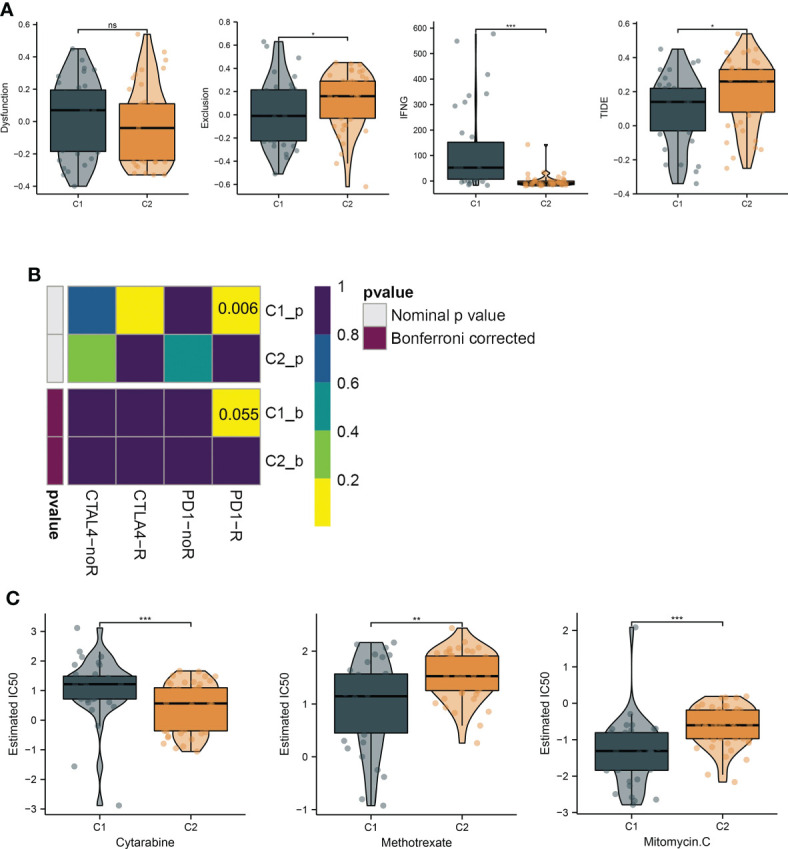
Hypoxia subtypes correlate with immunosuppression and their chemotherapeutic drugs development. **(A)** The TIDE estimation showing the dysfunction, exclusion, IFNG, and TIDE scores of the UM samples. **(B)** The submap of the expression similarity compared with immunotherapy response/nonresponse cohort. **(C)** Box plots present the IC50 differences of GDSC drugs between the subtypes. *, **, ***, and ns represents p-value<0.05, 0.01, 0.001, and not significant, respectively.

To investigate the immunological diversity between the clusters, we ran ssGSEA to analyze the immunocyte infiltration levels of each sample, and most of the immunocytes were highly infiltrated in cluster C1 (**Figure 2A**), including several immunosuppressive cells, such as regulatory T cells and myeloid-derived suppressor cells (MDSC). Further, we compared the expression of the immune checkpoint between clusters C1 and C2. Surprisingly, most of the immune checkpoints were upregulated in cluster C1 (**Figure 2B**). These results were quantified in box plots (**Figures 2C, D**) and demonstrated the immunosuppressive environment in high-hypoxia UM samples. Hence, we then conducted TIDE analyses. The results presented that cluster C1 exhibited a lower TIDE score, indicating that it may respond to immunotherapy (**Figure 3A**). We further analyzed the expression similarity between the training cohort and the previous immunotherapy cohort, and we noticed that cluster C1 samples showed similar expression signatures with the PD1-therapy response cohort though the *p*-value increased to 0.055 after Bonferroni correction (**Figure 3B**). Additionally, we also sought possible chemotherapeutic drugs for UM patients. The GDSC drug IC50 was predicted for each sample, and we obtained two drugs, Methotrexate and Mitomycin C, with lower IC50 in cluster C1 (**Figure 3C**). The immunotherapeutic analyses indicated that UM cancer with high hypoxia harbored immunosuppression and may benefit from immunotherapy.

### The clusters mainly differed in immune- and Extracellular matrix (ECM)-associated biological activities

**Figure 4:**
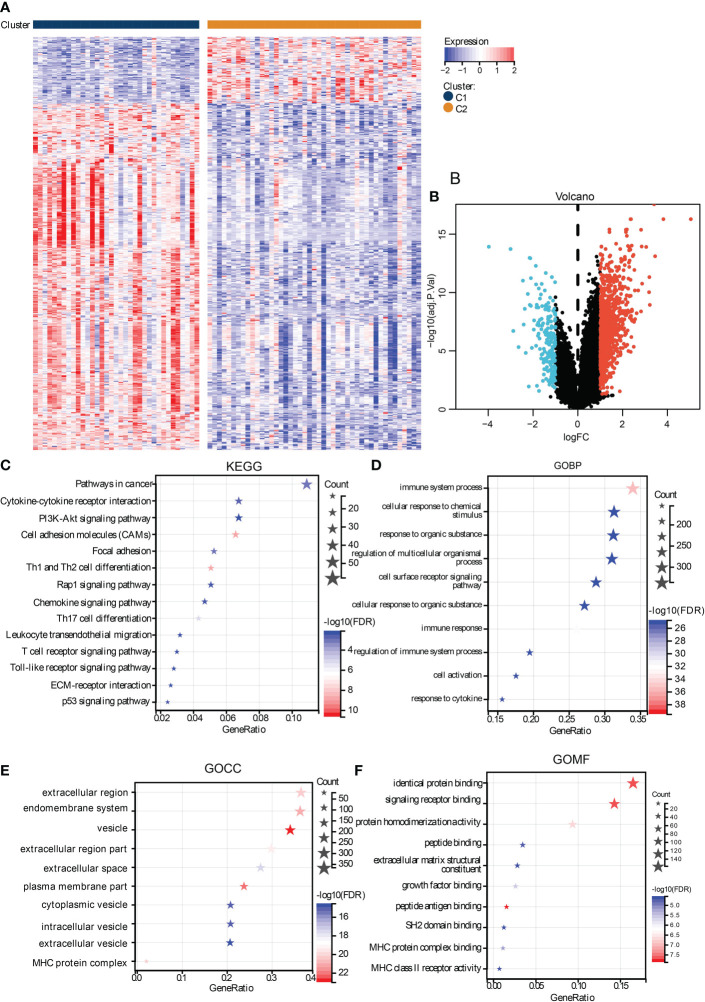
Functional analysis of DEGs between subtypes. The heat map **(A)** and volcano plot **(B)** shows the DEGs between subtypes. The functional analysis results were presented in bubble plots presenting the enriched KEGG **(C)**, GOBP **(D)**, GOCC **(E)**, and GOMF **(F)**, gene sets.

To investigate the biological activity differences between clusters, we first filter the DEGs between them, and many upregulated genes were identified in cluster C1 (**Figures 4A, B**). To annotate their function, we performed functional enrichment analyses by the KEGG and GO gene sets. The results exhibited that their DEGs were mainly enriched in immune-related pathways. For instance, the cytokine–cytokine receptor interaction, chemokine signaling pathways in KEGG, cellular response to cytokine in GOBP, MHC protein complex in GOCC, and GO MHC class II in GOMF were identified, similar to the immune analyses results. Besides this, ECM-associated pathways, such as the ECM-receptor interaction in KEGG, extracellular region in GOCC, and extracellular matrix structural constituent in GOMF, were also enriched (**Figures 4C–F**), demonstrating the close correlation between hypoxia and the microenvironment.

### Establishment of a hypoxia-based risk score

**Figure 5:**
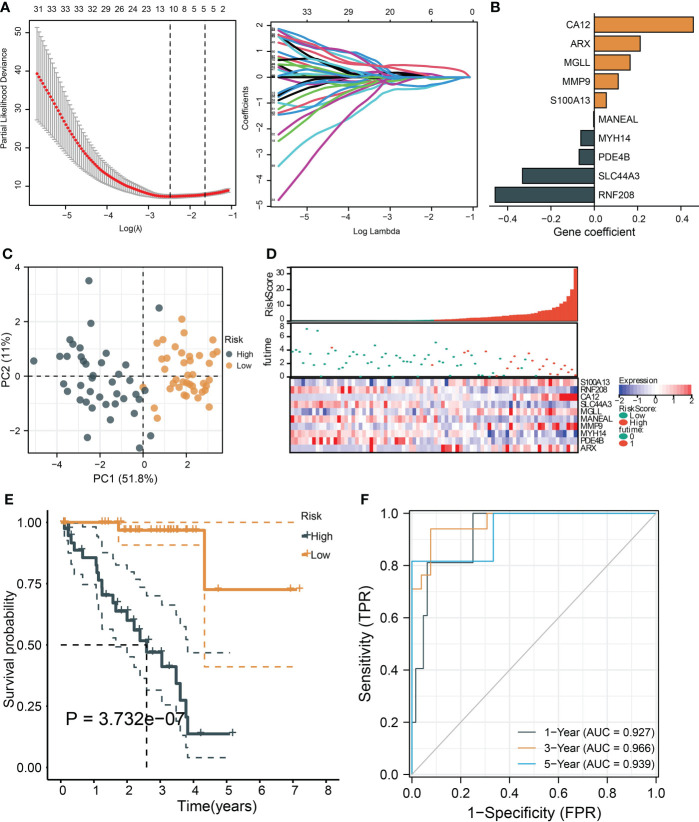
**|**Construction and validation of a hypoxia-based risk score. **(A)** The LASSO partial likelihood deviance plot and the coefficient profiles of all hypoxia gene. **(B)** The coefficients of the finally selected factors were presented in a bar chart. **(C)** The PCA plot shows the distance between the high- and low-risk group samples. **(D)** The risk survival table depicts the risk score gene expression pattern and patient survival status ranged by their risk score. **(E)** The Kaplan–Meier curve tests risk score’s ability to predict patient survival. **(F)** The ROC for estimating the model prediction accuracy of 1-, 3-, and 5-year survival.

**Figure 6:**
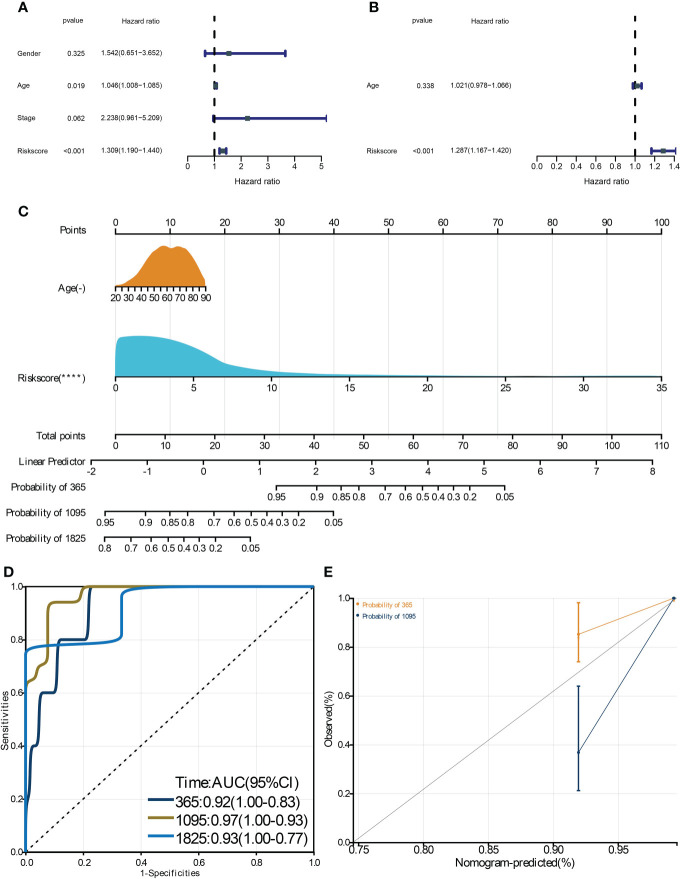
Construction and validation of a clinical nomogram. The univariate **(A)** and multivariate **(B)** Cox regression analysis to filter the prognostic predictors. **C:** The nomogram integrating the risk score and age to predict patient 1-, 3-, and 5-year survival. The ROC **(D)** and calibration curve **(E)** were used to evaluate the performances of the nomogram for predicting patient survival. **** means p-value < 0.0001.

To identify the critical genes in the effects of hypoxia, we used the hypoxia gene set to establish a risk score. The LASSO algorithm reduced the gene number to 10, and five risky and five protective genes were finally obtained with their coefficients, respectively (**Figures 5A, B**). The patients were divided into the high- and low-risk groups according to the median risk score of the training cohort. The PCA plot showed that the risk groups separated obviously (**Figure 5C**). The risk-survival plot exhibited that the five risk genes’ expression elevated as the risk score increased, and the opposite trend was observed for the five protective genes (**Figure 5D**). For the prognostic value, the high-risk group patients suffered significantly lower survival rates, and the ROC results demonstrated the risk score predicted patient overall survival with a high accuracy (**Figures 5E, F**). When validated in the validating cohorts, the same expression trend of the 10 genes was observed, and the risk score can predict the patient survival with high accuracy (Supplementary Figures S1A–S1F). We performed univariate and multivariate Cox analysis to select the prognostic clinical predictors apart from the risk score and age, and risk score passed the univariate test though it failed in the multivariate test (**Figures 6A,B**). When integrating the risk scores and age to form a nomogram, the nomogram also presented good performance in predicting patient survival (**Figures 6C–E**).

### The risk groups presented diverse immunological characteristics and sensitivity to some newly identified drugs

**Figure 7:**
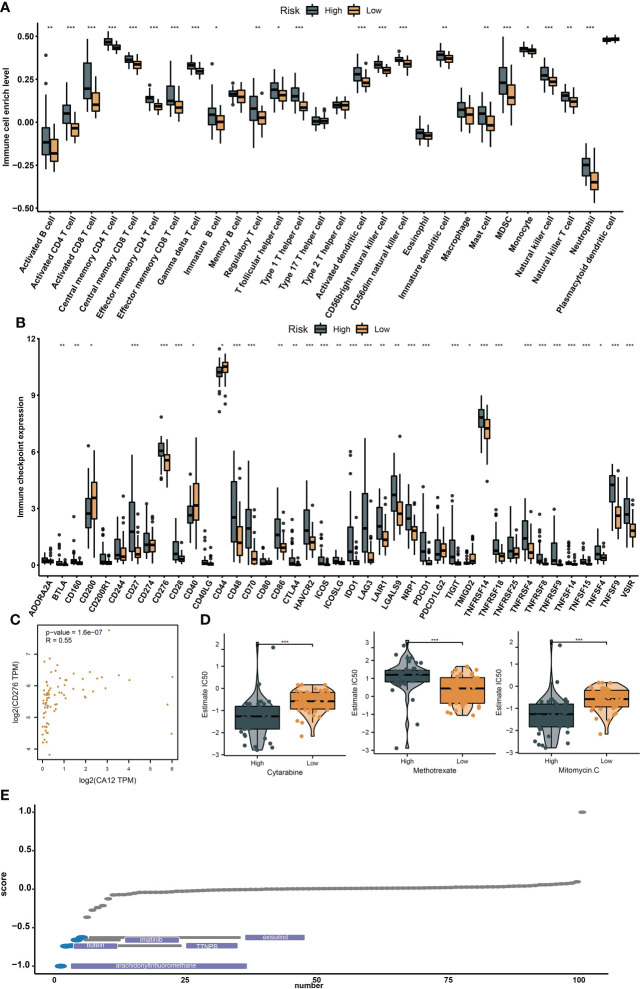
The immunological diversity between the risk groups and drug development. The box plots show the infiltration differences of immunocytes **(A)** and immune checkpoint differences **(B)** between the risk groups. **(C)** The correlation between CD12 and CD276 expression. **(D)** The IC50 level differences between the risk groups for the GDSC drugs. **(E)** Identification of potential effective drugs from the cMap database, *, **, *** represents p-value < 0.05, 0.01, and 0.001, respectively.

We performed ssGSEA to compare the differences of immunocyte infiltration between the risk groups, and we noticed that many immunocytes were highly infiltrated in the high-risk group, including the immunosuppressive cells (regulatory T cell and MDSC) (**Figure 7A**). Also, the majority of the immune checkpoints were upregulated in the high-risk group, including CD276, CTLA4, and PDCD1, et al. (**Figure 7B**). We then searched GEPIA2.0 and discovered that CD276 and CA12 were significantly and positively correlated as their correlation coefficient reaches 0.55 (**Figure 7C**). Finally, we identified two drugs from GSDC and five drugs from cMap with therapeutic potential for high-risk patients (**Figures 7D, E**).

### The risky genes were upregulated under hypoxia and CA12-knockdown affects EMT, cell cycle, and immune checkpoint expression

**Figure 8:**
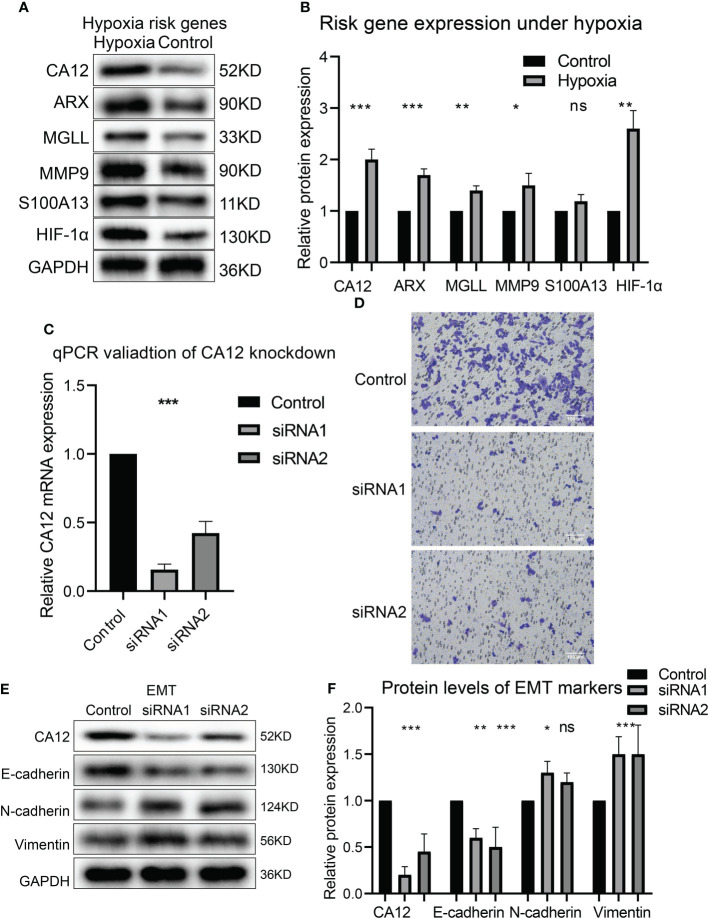
The risk gene expressions under hypoxia and the role of CA12 in UM cell EMT process. The protein bands of the risk gene expressions under normal and hypoxia conditions **(A)** and the quantification of the protein expression results in the box plot **(B)**. **(C)** The statistical comparison of the RT-qPCR results. **(D)** The image of transferred cells in control, CA12 knockdown-siRNA1, and siRNA2 groups. The bands of EMT pathway protein expressions **(E)** and their quantification in the box plots **(F)** between control, siRNA1, and siRNA2 groups, ns, *, **, *** represents not statistically significant, p-value < 0.05, 0.01, and 0.001, respectively.

To experimentally validate whether the risky genes were correlated with hypoxia, we performed hypoxia cultivation, and we noticed that CA12, ARX, MGLL, and MMP9 were significantly upregulated under hypoxia; S100A13 was not significantly upregulated but also showed a similar trend (**Figures 8A, B**). Subsequently, we analyzed the effects of the top risky gene CA12 knockdown on cell phenotypes. The RT-qPCR results validated that the CA12 mRNA levels decreased significantly in both CA12-knockdown groups (**Figure 8C**). The Transwell results demonstrated that knockdown of CA12 significantly inhibited cell migration (**Figure 8D**), indicating a depressed EMT processes. Hence, we detected the E-cadherin, N-cadherin, and Vimentin protein expressions, representing the EMT process; the upregulated Vimentin, N-cadherin, and downregulated E-cadherin suggested an activated EMT process of MUM2B cells to enhance their migration though the N-cadherin upregulation of the siRNA-2 group was not statistically significant (**Figures 8E, F**).

**Figure 9:**
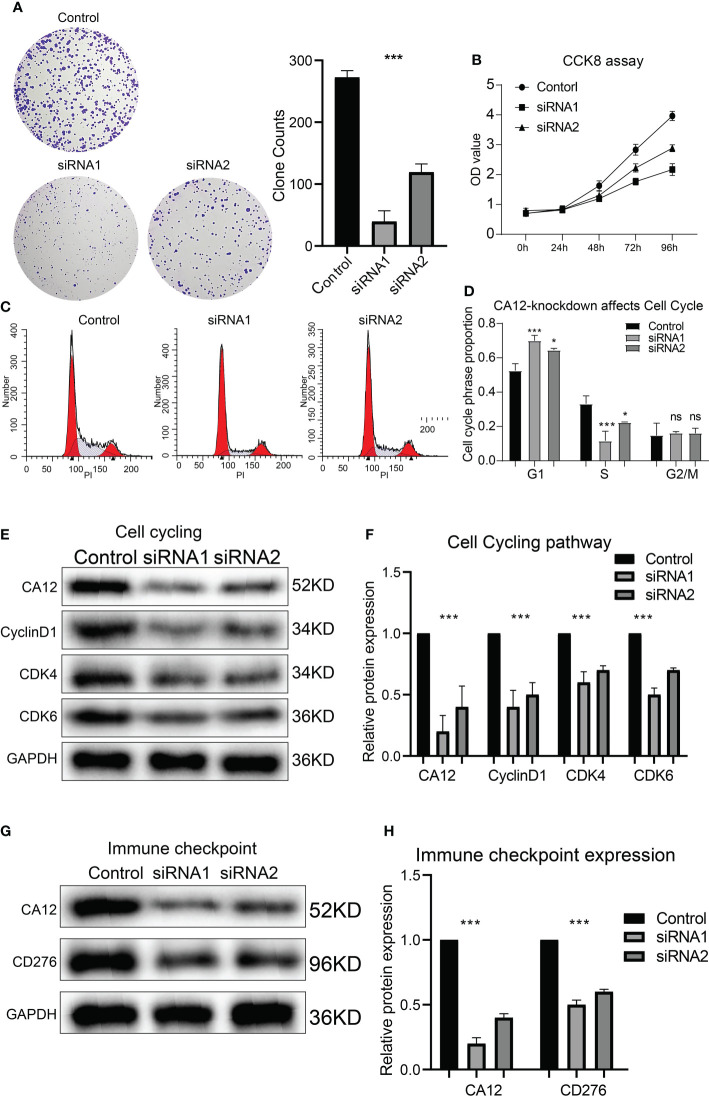
Roles of CA12 knockdown in UM cell growth and immune checkpoint expression. The images and the quantification results of clone formation assay **(A)** and the results of CCK8 assay **(B)** for the CA12-knockdown experiments. The cell cycle flow cytomatrix results **(C)** and the comparison of G1, S, and G2/M phases proportion **(D)** between control and siRNA-knockdown groups. The bands of G1 phase protein expressions **(E)** and their quantification comparison **(F)** between control and siRNA-knockdown groups. The bands of CD276 protein expression **(G)** and its quantification comparison **(H)** between control and siRNA-knockdown groups ns, *, *** means not statistically significant, p-value<0.05, and 0.001, respectively.

Besides this, the clone formation and CCK8 assay results presented that CA12 knockdown also depressed cell viability and clone-formation ability (**Figures 9A, B**). Further, we performed flow cytometric analysis to investigate whether CA12 knockdown affected the cell cycle cells, and we found that CA12 knockdown increased the proportion of the G1 phase and decreased the S phase of cells (**Figures 9C, D**), implying the G1 phase arrest in MUM2B cells. The G1 phase–related proteins (cyclinD1, CDK4, and CDK6) were detected, and the decreased cyclinD1, CDK4, and CDK6 were observed in the CA12-knockdown group (**Figures 9E, F**), demonstrating that CA12 knockdown induced the G1 phase arrest.

Finally, we explored whether CA12 was associated with immune checkpoints. We detected the protein expression of CD276, and the results exhibited that knockdown of CA12 significantly decreased the expression of CD276 (**Figures 9G, H**). These results manifested that CA12 was a critical risky factor of UM for its association with EMT, cell cycle, and immune checkpoint CD276.

## Discussion

Hypoxia has various effects on cancer progression and correlates with multiple cancer hallmark features (8). In UM, hypoxia also affects cancer cell behavior like angiogenesis (11), but the extensive mechanism remained unknown, such as its interaction with cancer immunity. Also, hypoxia-based therapeutic research, except for HIF-targeted treatment, is still empty. Here, we identified hypoxia-related subtypes and constructed a hypoxia-related risk score for patient survival prediction. The subtypes and the risk score can significantly separate the patients’ survival rates, and particularly, the risk score predicted patient overall survival with a high accuracy in both the training and validating cohorts according to a criteria for prediction models (20), indicating that our model was well-designed with critical hypoxia factors in cancer cell fate. To our knowledge, this is the first model focusing on the hypoxia signature’s influence on UM patient prognosis.

During the construction of the hypoxia model, we identified 10 prognostic genes, including five risk and five protective factors. Among the 10 key genes, CA12, MMP9, SLC44A3, and RNF208 have been reported to associate with UM patient survival. Carbonic anhydrase 12 (CA12) belongs to the zinc metalloenzymes that catalyzed the carbon dioxide reversible hydration. The role of CA12 in cancers remained paradoxical for it promoted pancreatic cancer apoptosis (21) while accelerating the EMT progression of glioma (22), suggesting its complex effects on cancers. In our study, we identified it as a risk gene and confirmed that CA12 knockdown arrests the cell cycle and inhibited the EMT transformation of UM cells; this is novel in UM study since no reports concerning the role of CA12 in UM have been reported. MMP9 is a matrix metalloproteinase, which degrades the extracellular matrix proteins. MMP9 has been discovered as a risk gene and predicts a worse prognosis for UM patients (23). SLC44A3 and RNF208 were found to be protective predictors (24); these discoveries are consistent with our findings in this study. Besides this, the other six genes (ARX, MGLL, S100A13, MANEALM, MYH14, and PDE4B) have not been presented in UM so far, and we first identified these genes as novel prognostic factors in UM tumors.

Hypoxia has been suggested to affect immunotherapy by tumor cell anaerobic glycolysis; the metabolite adenosine secreted to the ECM suppressed T cell activation, and thus, excused the tumor cells from immune attack (25), indicating the promising therapeutic strategy developed from cancers with hypoxia. Currently, hypoxia has presented association with immunotherapy response or immune checkpoint effects in many cancers (26–28), whereas only a clinical trial consisting of mixed melanoma patients suggested that the PD-1 blockage responders of immunotherapy has a reduced hypoxia transcriptomic change (29). In this study, we observed many upregulated immune checkpoints in the high-risk group and C1 subtype and transcription similarity with the samples that responded to the PD-1 therapy, demonstrating the potential of hypoxia as the target to improve immunotherapeutic effects. Most importantly, we validated that CD276 was downregulated when CA12 knockdown was conducted. CD276 was first derived from dendritic cells and the immune checkpoint in cancers; it impaired T cell–mediated anticancer immunity in ovarian cancer and destroyed the anti-PD-1 therapy in non-small cell lung cancer (30). Whereas in UM, no study has been reported. Hence, we first identified the relationship between CD276 and hypoxia and suggested that targeting CA12 may be a potential approach to restore CD276-mediated immunotherapeutic effects. Moreover, elevated regulatory T cell and MDSC were observed in the high-risk group and C1 subtype since CD276 was expressed on regulatory T cells and MDSC (31, 32); this indicated the involvement of MDSC or regulatory T cells in CD276-mediated immunosuppression in UM.

Comprehensively, our study identified hypoxia-related UM subtypes and risk groups, which accurately predicted the UM patient prognosis. The subtype or risk group with high hypoxia signature expression exhibited highly infiltrated immunocytes and immune checkpoints and presented transcriptional similarity with those responding to PD-1 therapy. Further, we confirmed the upregulation of the risk gene under hypoxia and validated that knockdown of CA12 inhibited UM cell EMT, clone formation, and G1/S phase transformation. Besides this, the CD276 expression decreased with CA12 knockdown. This study discovered the association between hypoxia and cancer immunity in UM and will shed light on novel therapeutic strategies development.

## Data availability statement

The original contributions presented in the study are included in the article/**Supplementary Material**. Further inquiries can be directed to the corresponding author.

## Ethics statement

Ethical review and approval was not required for the study on human participants in accordance with the local legislation and institutional requirements. Written informed consent from the patients/participants or patients/participants’ legal guardian/next of kin was not required to participate in this study in accordance with the national legislation and the institutional requirements.

## Author contributions

FL contributes to the data collection, concept, and study design; YY and WD performed the data analysis and wrote the first manuscript draft. All authors approved the submission of this manuscript.

## Conflict of interest

The authors declare that the research was conducted in the absence of any commercial or financial relationships that could be construed as a potential conflict of interest.

## Publisher’s note

All claims expressed in this article are solely those of the authors and do not necessarily represent those of their affiliated organizations, or those of the publisher, the editors and the reviewers. Any product that may be evaluated in this article, or claim that may be made by its manufacturer, is not guaranteed or endorsed by the publisher.

## Abbreviations

UM, Uveal melanoma; GO, Gene ontology; KEGG, Kyoto Encyclopedia of Genes and Genomes; TCGA, The Cancer Genomes Atlas; GEO, Gene Expression Omnibus; TIDE, Tumor Immune Dysfunction and Exclusion; GSVA, Gene Set Variation Analysis; PCA, Principal component analysis; GSEA, Gene Set Enrichment Analysis; DEGs, Differentially expressed genes; BP, biological process;, CC, cellular component;, and MF, molecular function; LASSO, Least absolute shrinkage and selection operator; GDSC, Genomics of Drug Sensitivity in Cancer; PI, propidium iodide; ROC, Receiver operating characteristics curve; MDSC, Myeloid-derived suppressor cells; ECM-associated, Extracellular matrix; CA12, Carbonic Anhydrase 12.
