# Supplementary material for: The construction of a hypoxia-based signature identified CA12 as a risk gene affecting uveal melanoma cell malignant phenotypes and immune checkpoint expression
Source: Front Oncol. 2022 Sep 26;12:1008770. doi: 10.3389/fonc.2022.1008770 (PMC9548707; doi:10.3389/fonc.2022.1008770)

consensus matrix legend

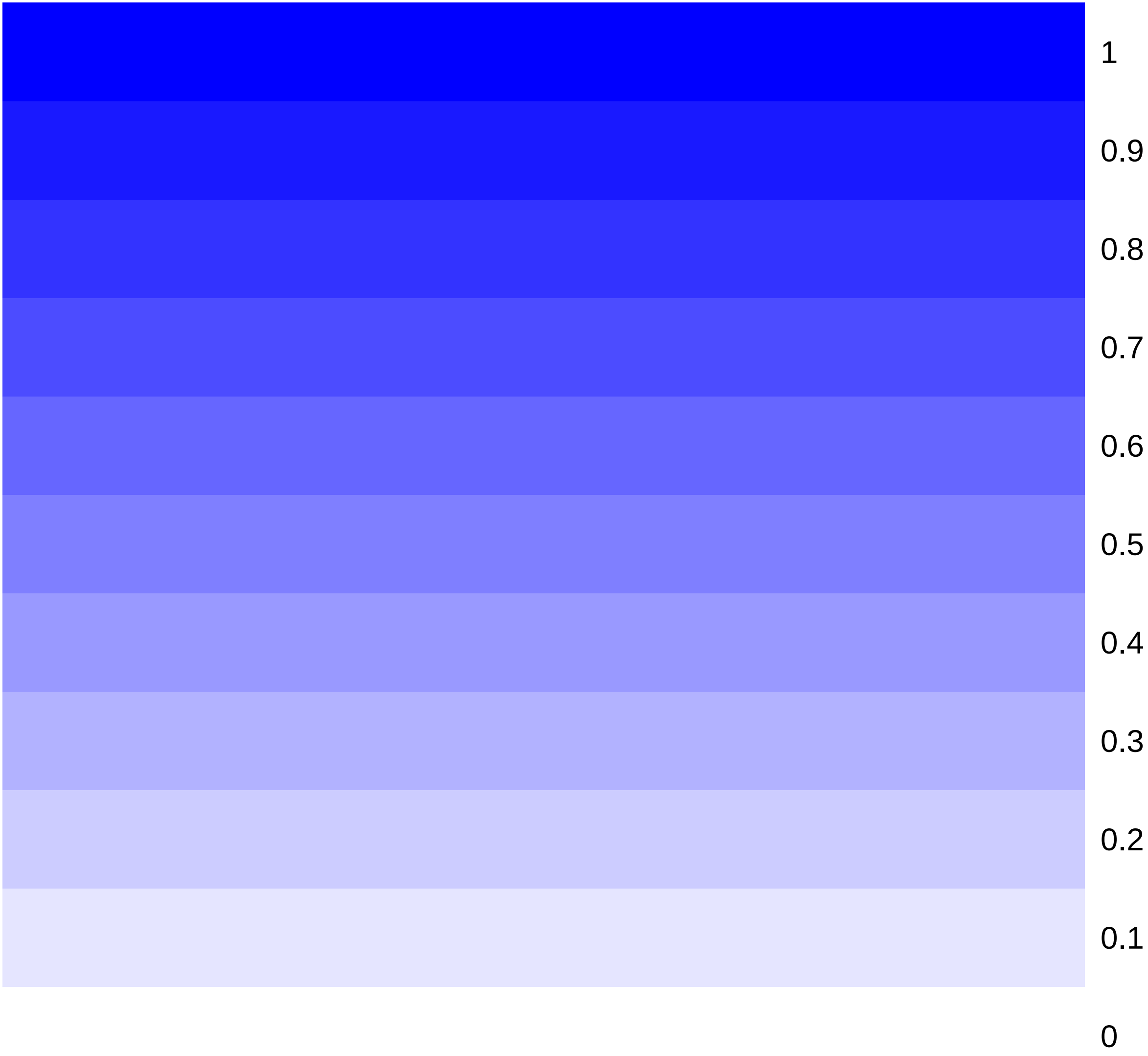

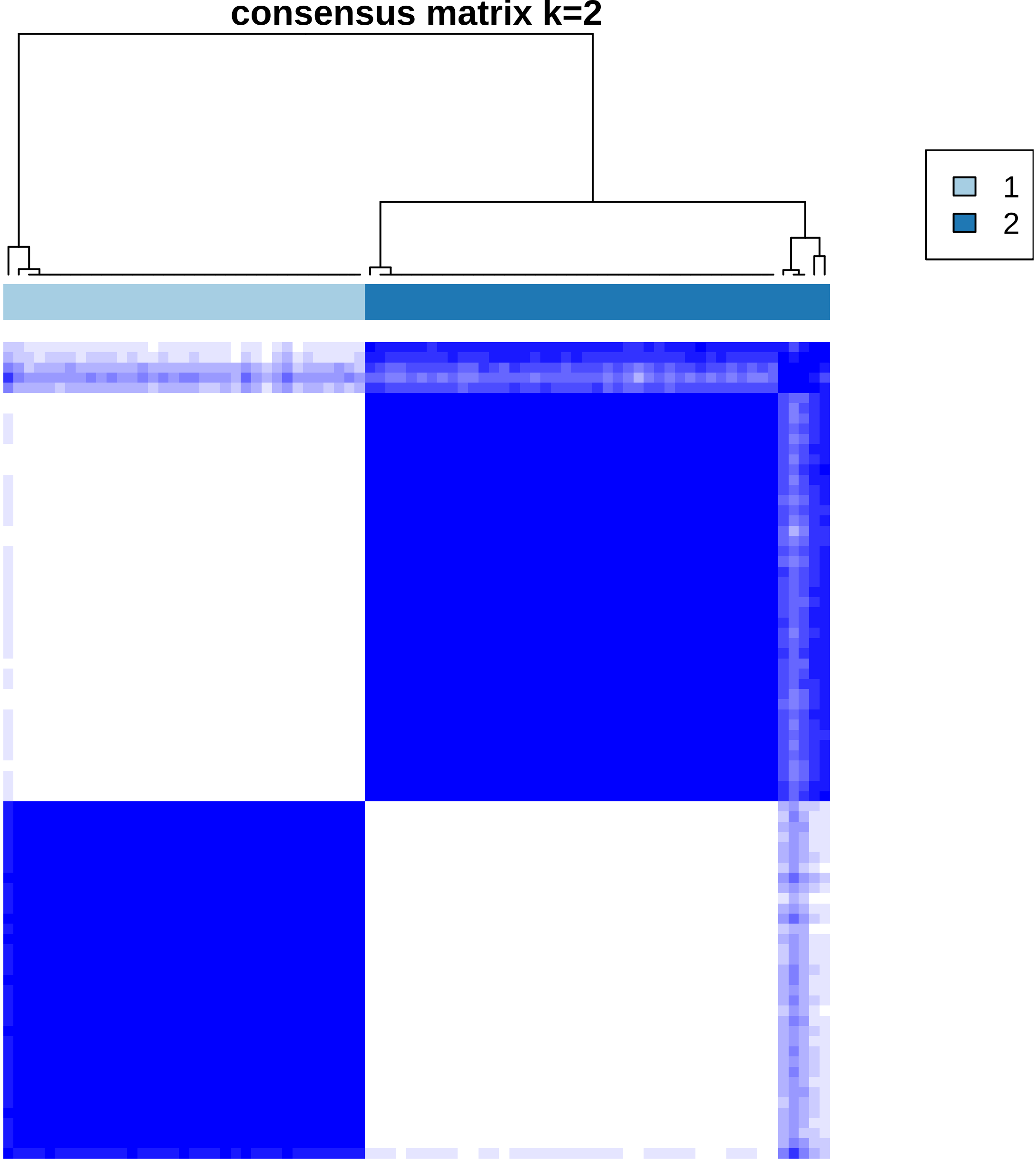
consensus matrix k=2
1
2

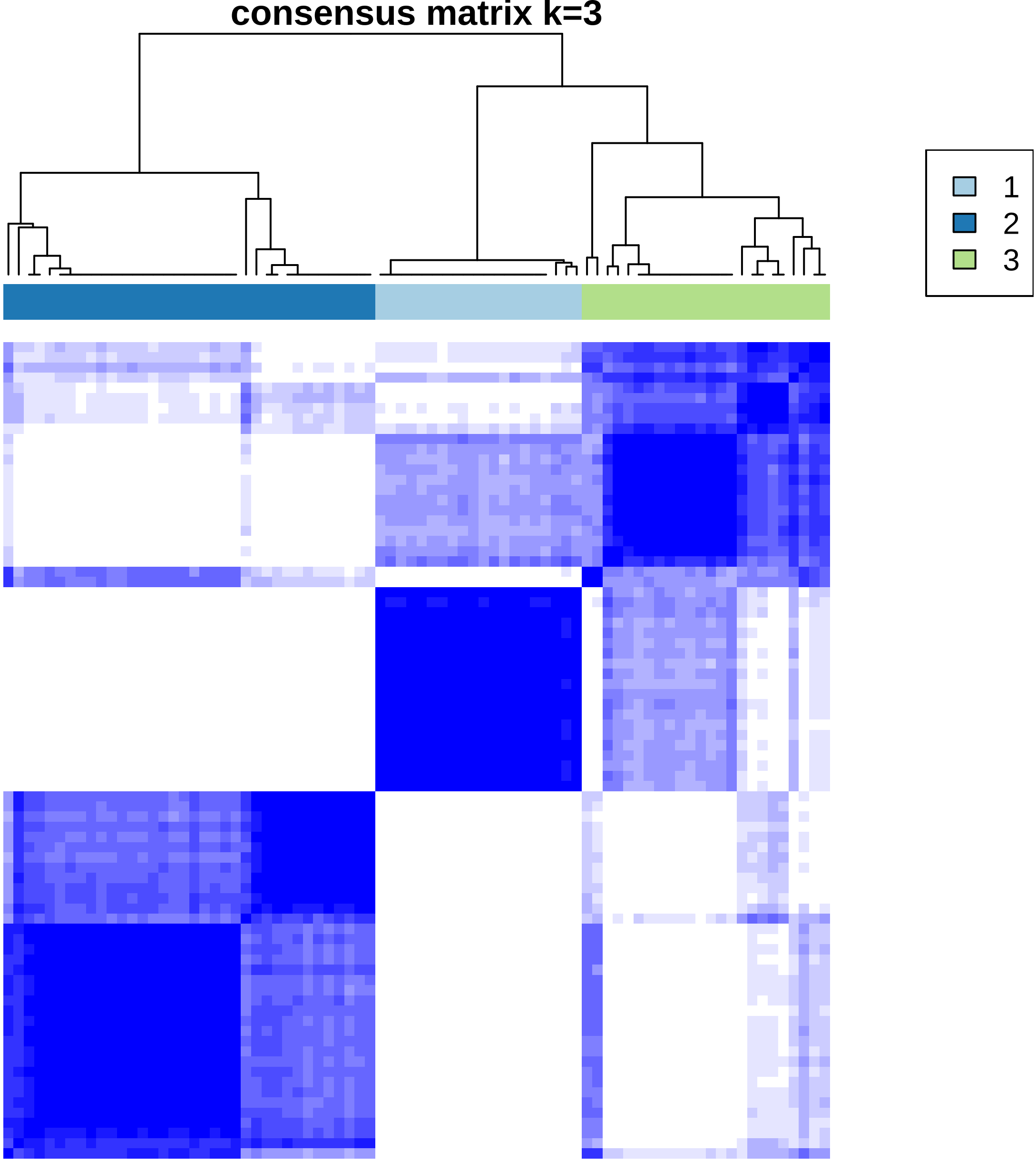
consensus matrix k=3
1
2
3

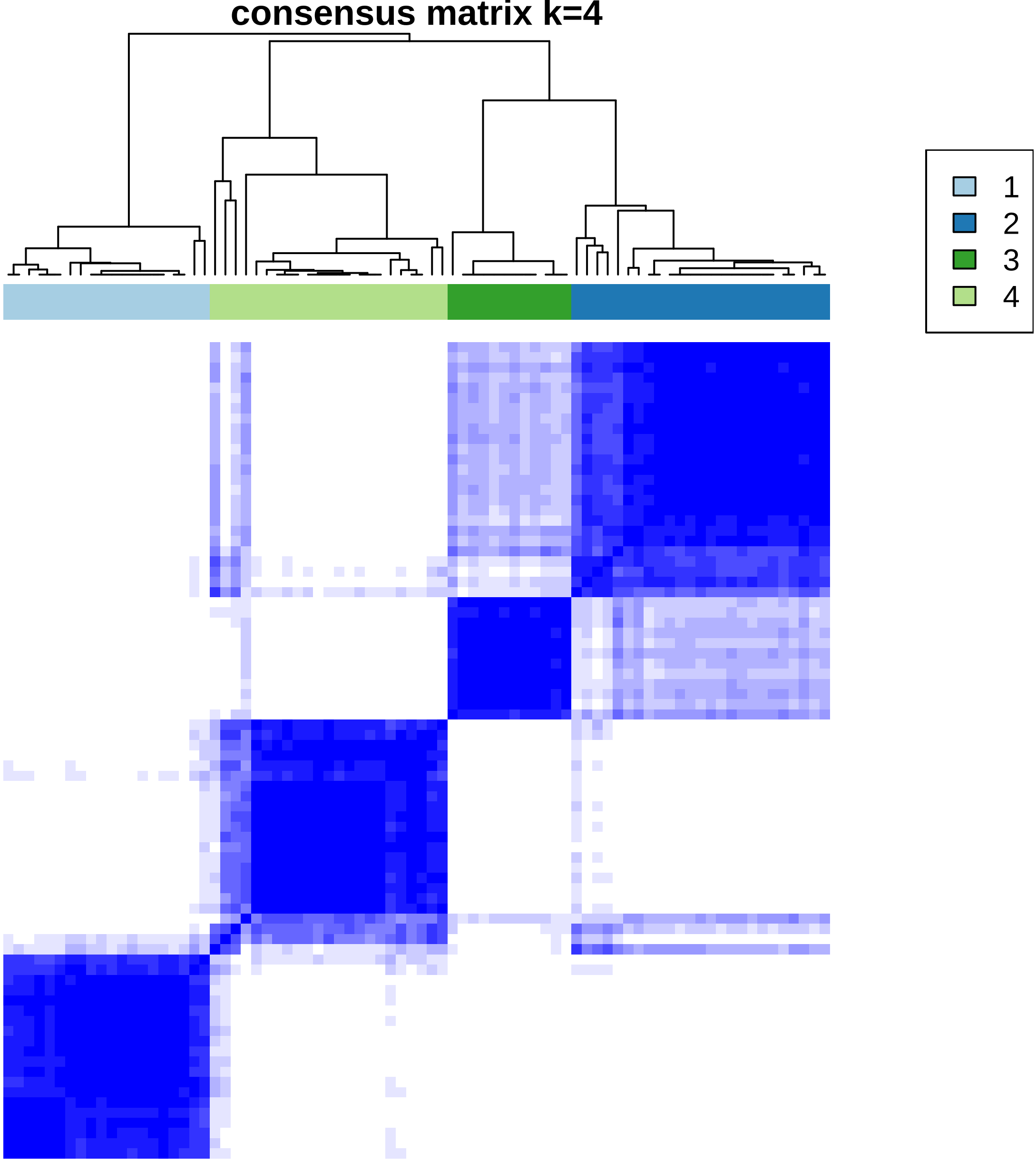
consensus matrix k=4
1
2
3
4

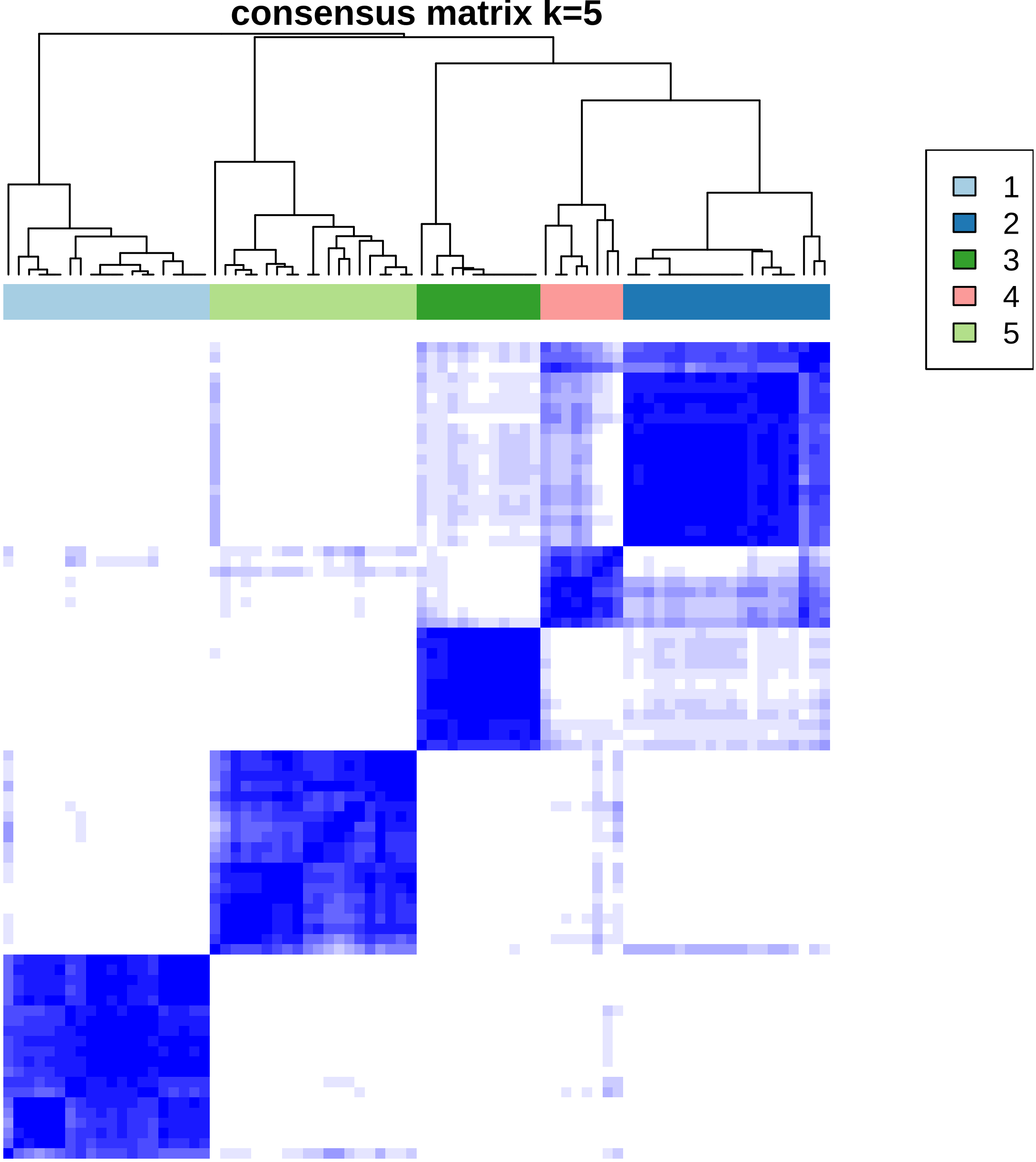
consensus matrix k=5
1
2
3
4
5

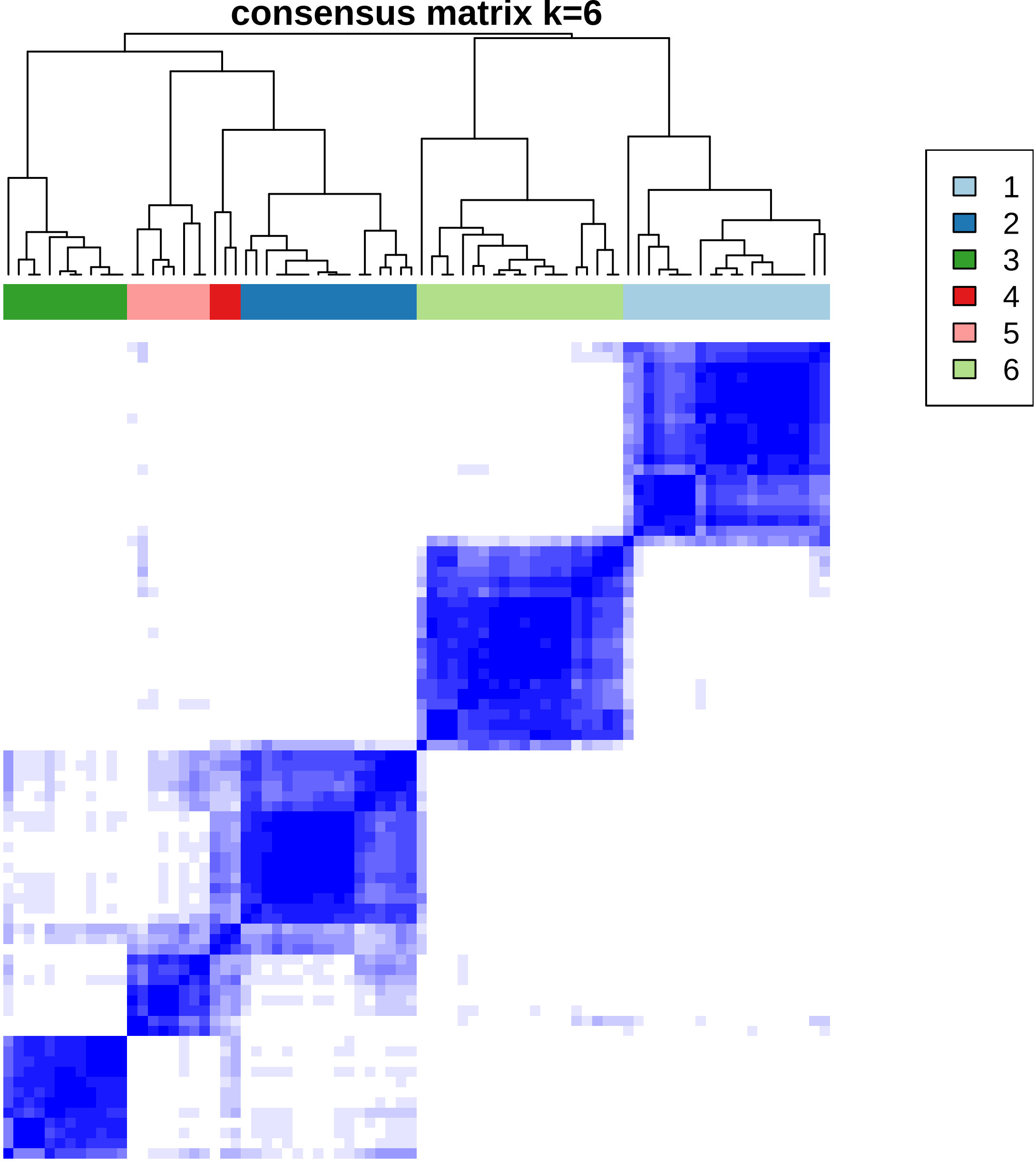

consensus matrix k=6
1
2
3
4
5
6

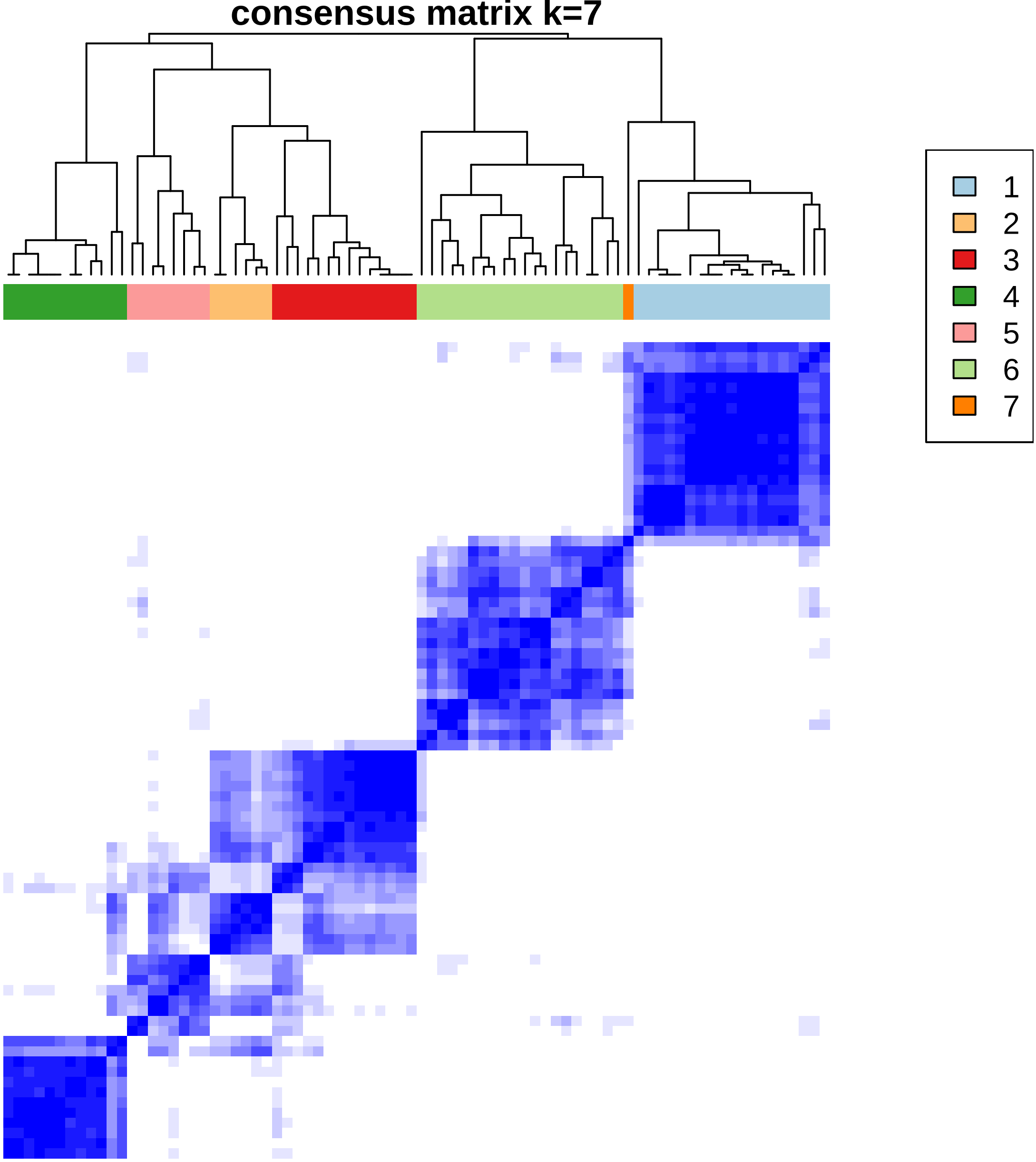
consensus matrix k=7
1
2
3
4
5
6
7

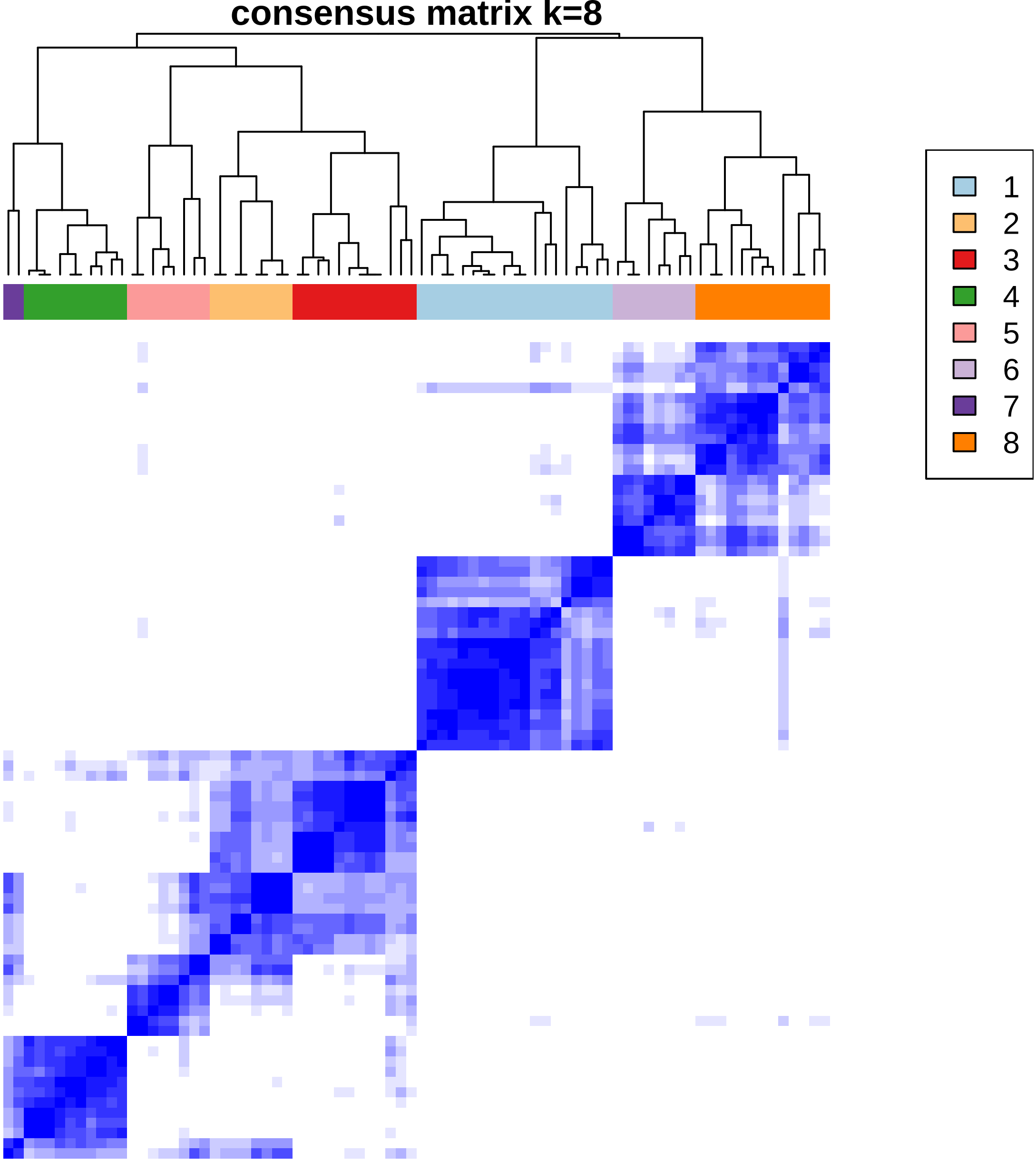
consensus matrix k=8
1
2
3
4
5
6
7
8

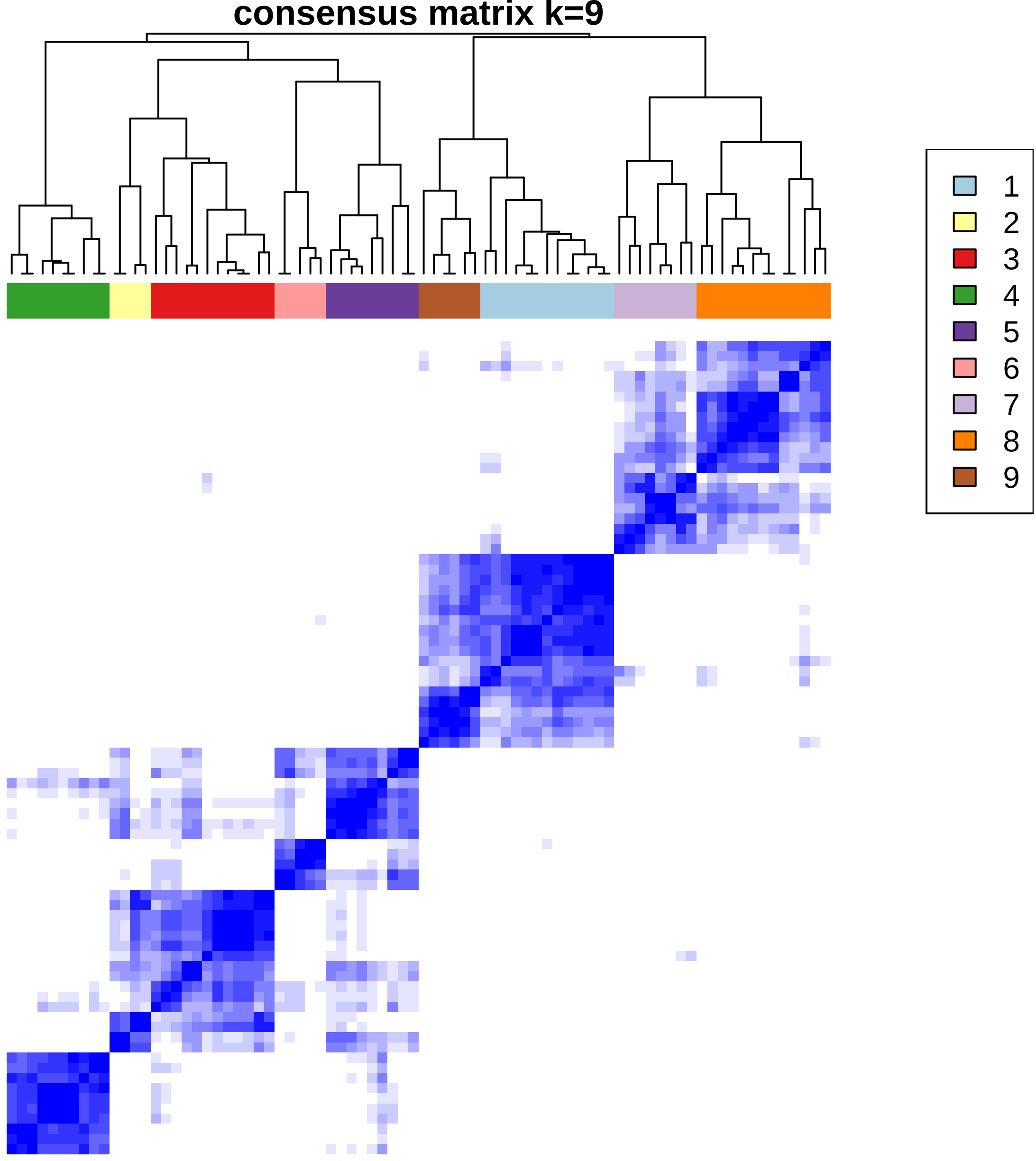
consensus matrix k=9
1
2
3
4
5
6
7
8
9

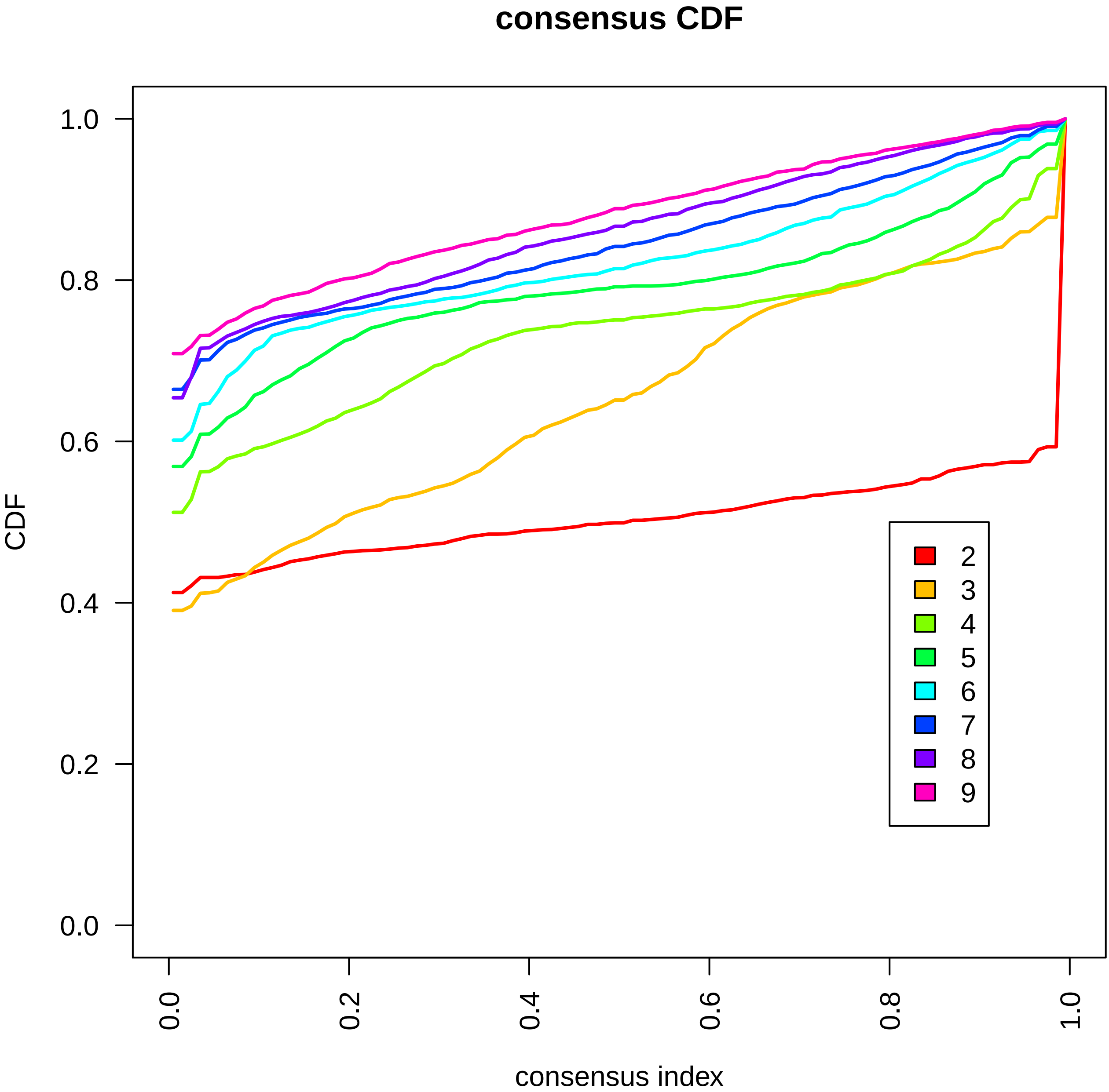
consensus CDF
CDF
consensus index
1.0
0.8
0.6
0.4
0.2
0.0
0.0
0.2
0.4
0.6
0.8
1.0
2
3
4
5
6
7
8
9

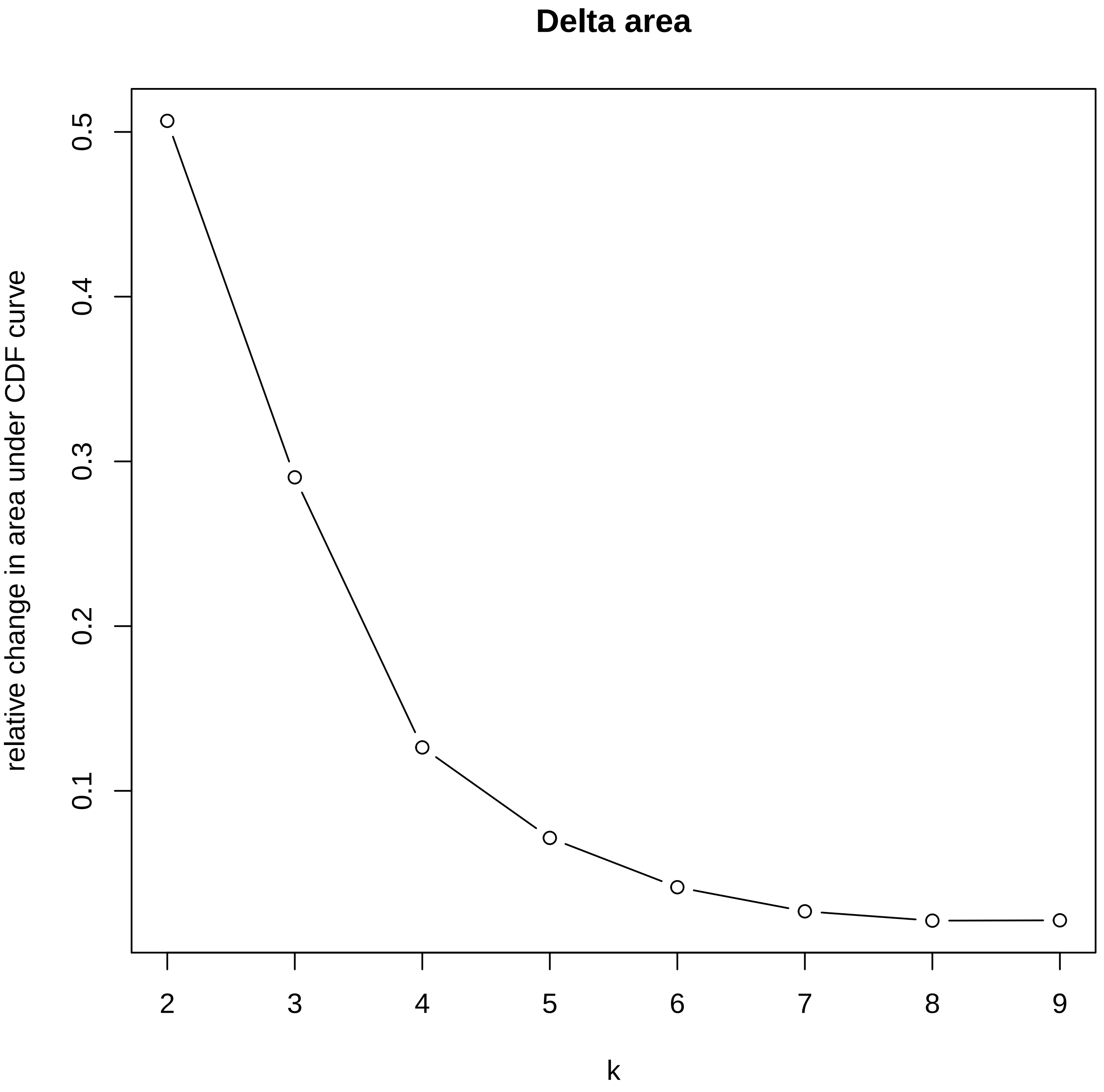
Delta area
relative change in area under CDF curve
0.1
0.2
0.3
0.4
0.5
2
3
4
5
6
7
8
9
k

tracking plot

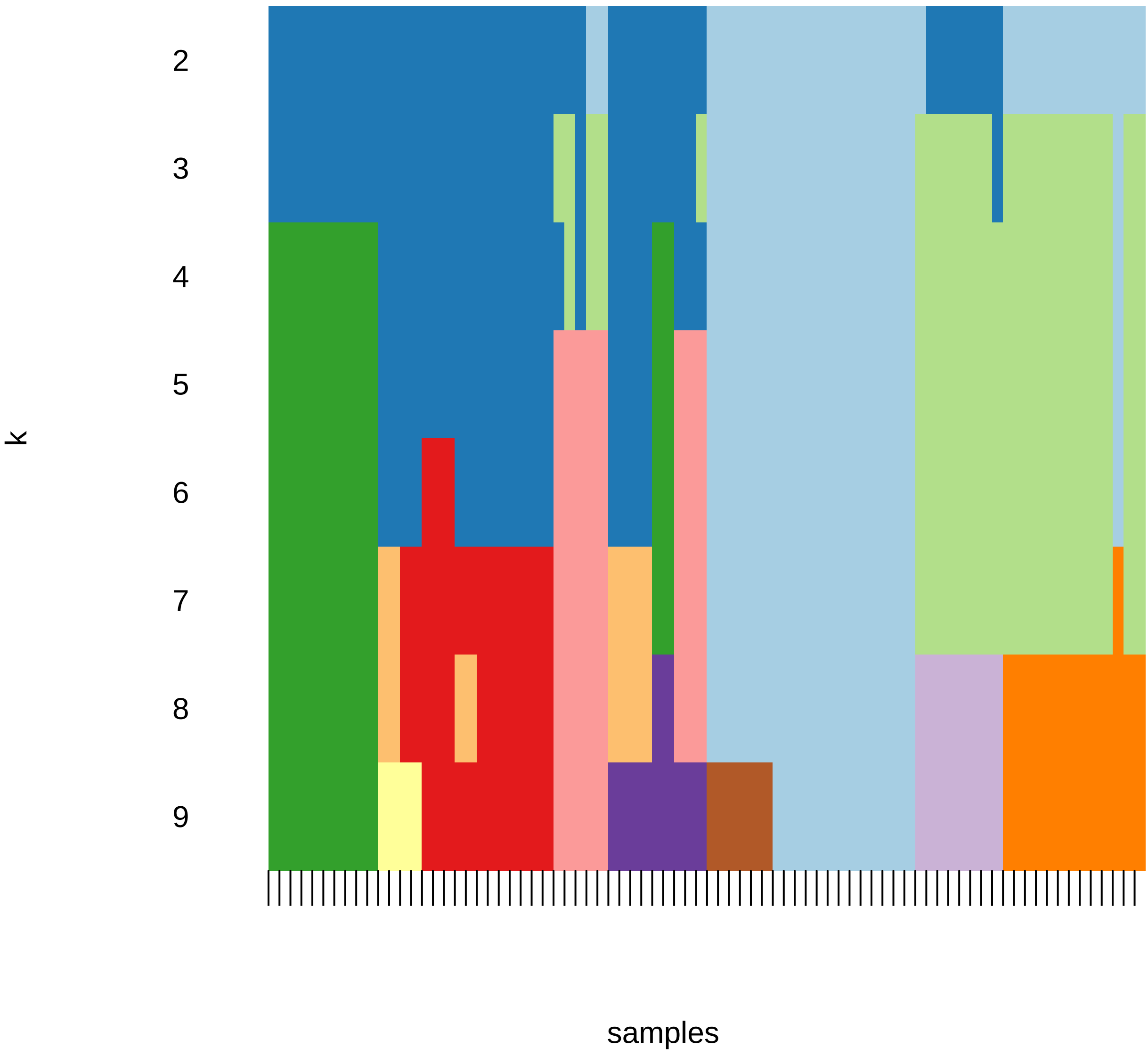

Supplement: Supplementary file 1 [file DataSheet_1.zip › Figure 1/consensus.pdf]
