# Supplementary figures and images for: The construction of a hypoxia-based signature identified CA12 as a risk gene affecting uveal melanoma cell malignant phenotypes and immune checkpoint expression
Source: Front Oncol. 2022 Sep 26;12:1008770. doi: 10.3389/fonc.2022.1008770 (PMC9548707; doi:10.3389/fonc.2022.1008770)

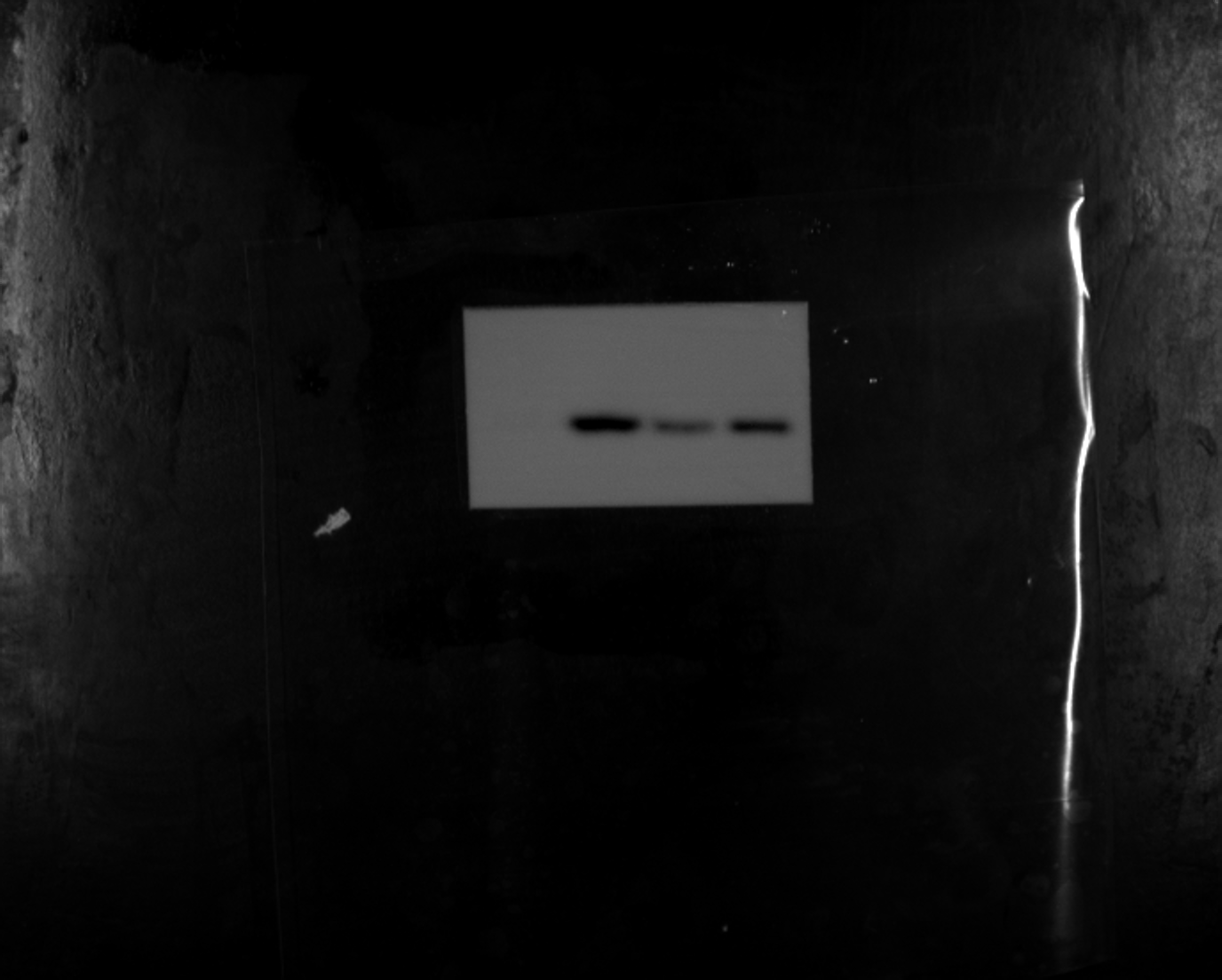

Supplement: Supplementary file 2 [file DataSheet_2.zip › Figure 8/EMT western blot/EMT_CA12.Tif]

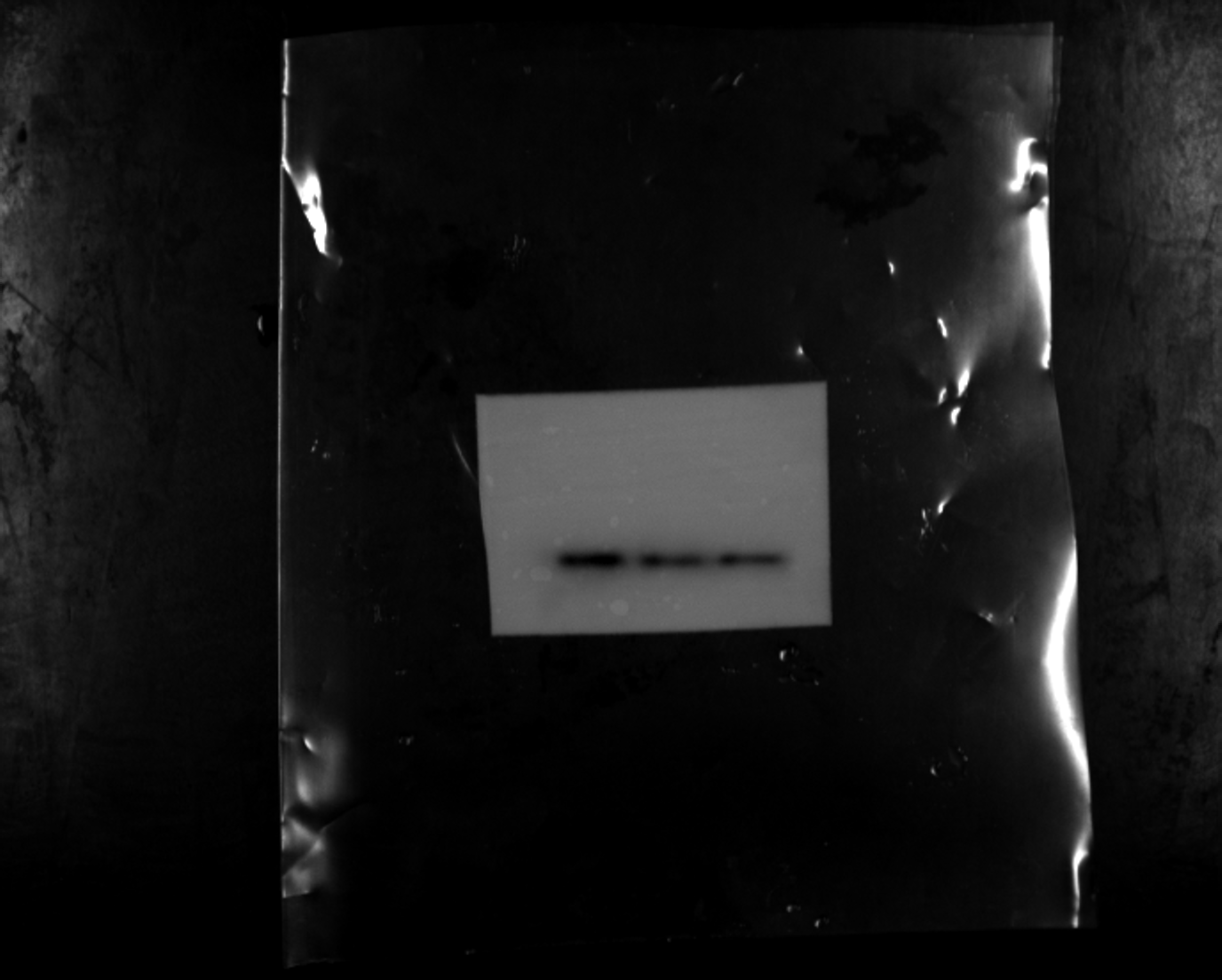

Supplement: Supplementary file 2 [file DataSheet_2.zip › Figure 8/EMT western blot/EMT_E-cadherin.Tif]

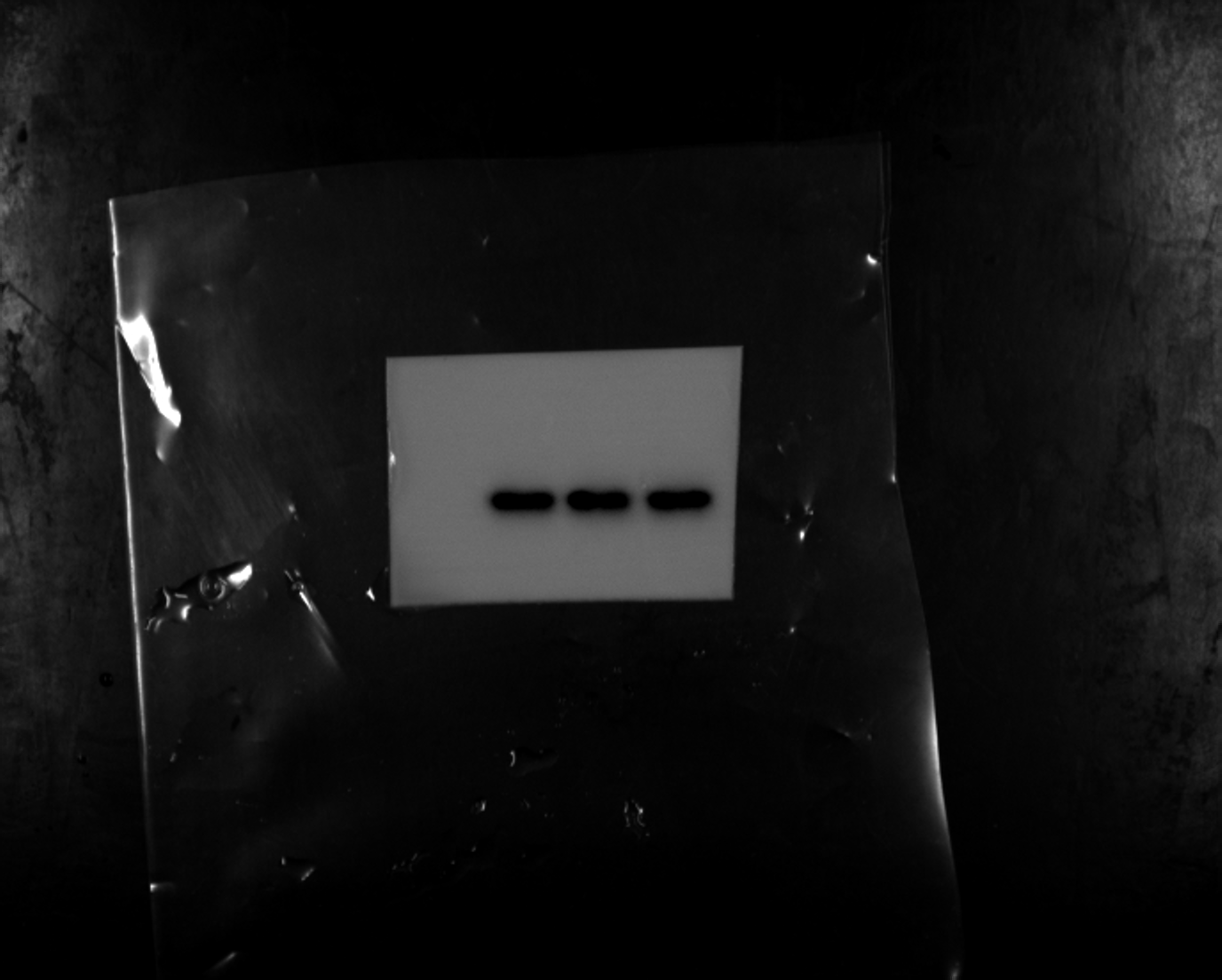

Supplement: Supplementary file 2 [file DataSheet_2.zip › Figure 8/EMT western blot/EMT_GAPDH.Tif]

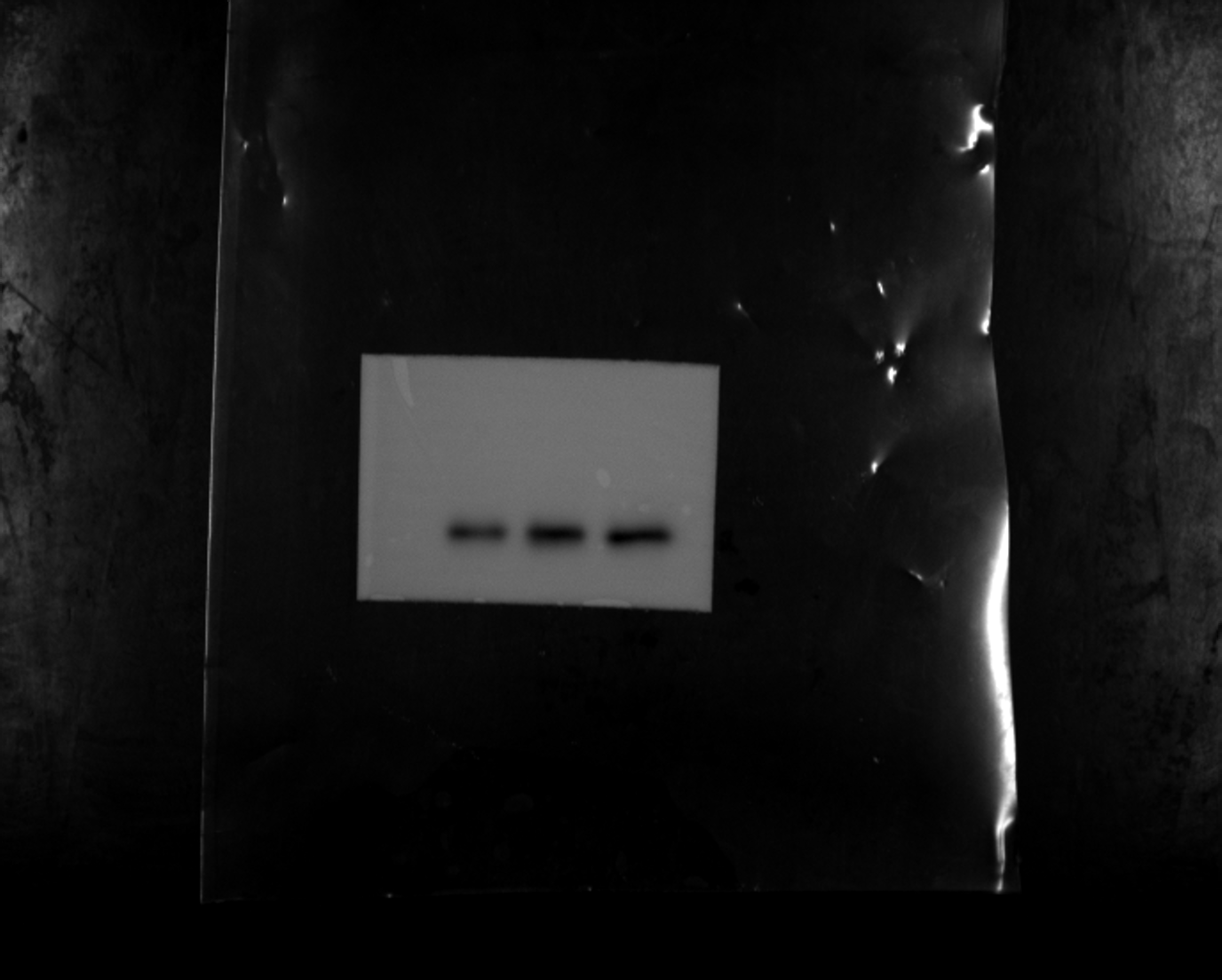

Supplement: Supplementary file 2 [file DataSheet_2.zip › Figure 8/EMT western blot/EMT_N-cadherin.Tif]

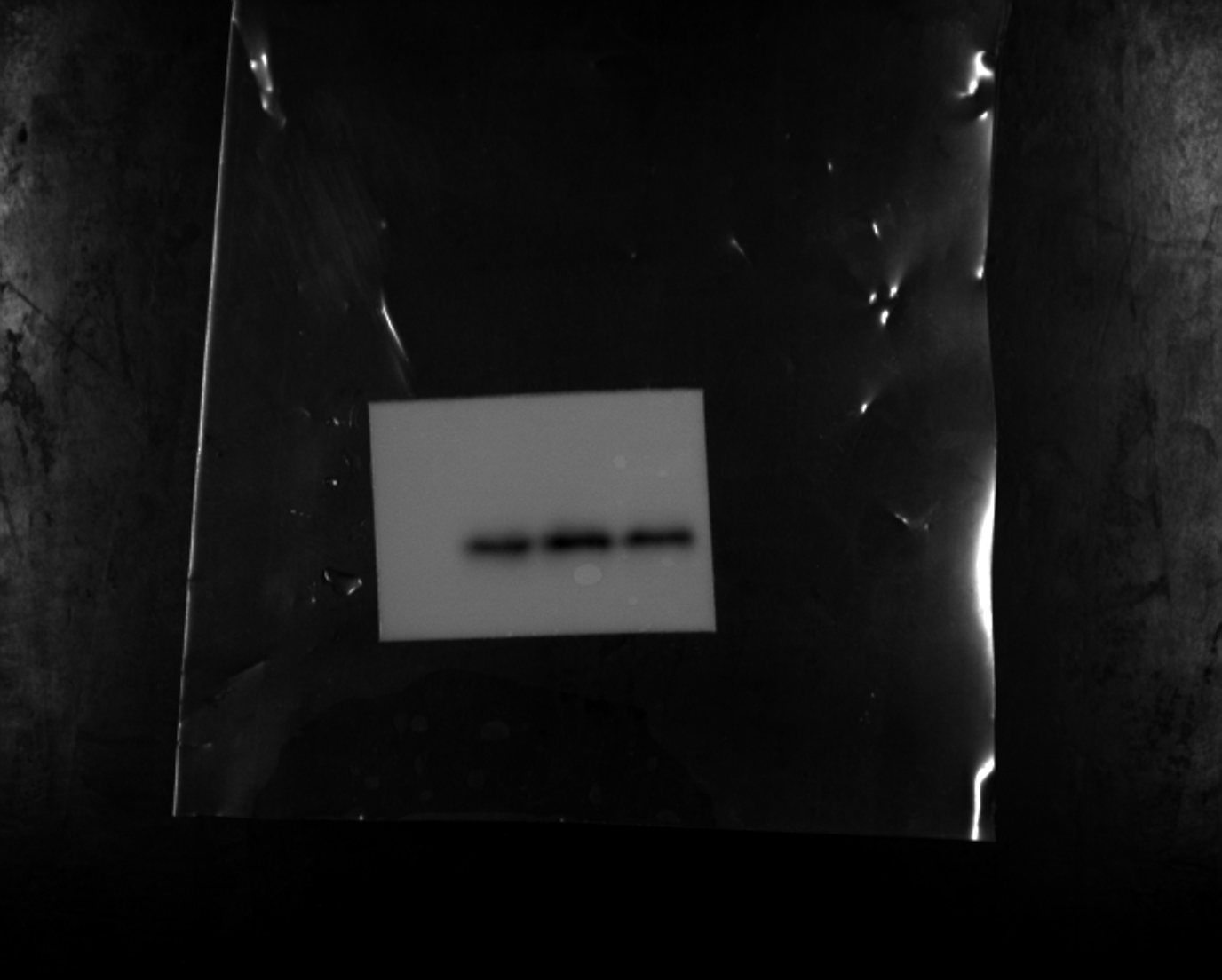

Supplement: Supplementary file 2 [file DataSheet_2.zip › Figure 8/EMT western blot/EMT_Vimentin.Tif]

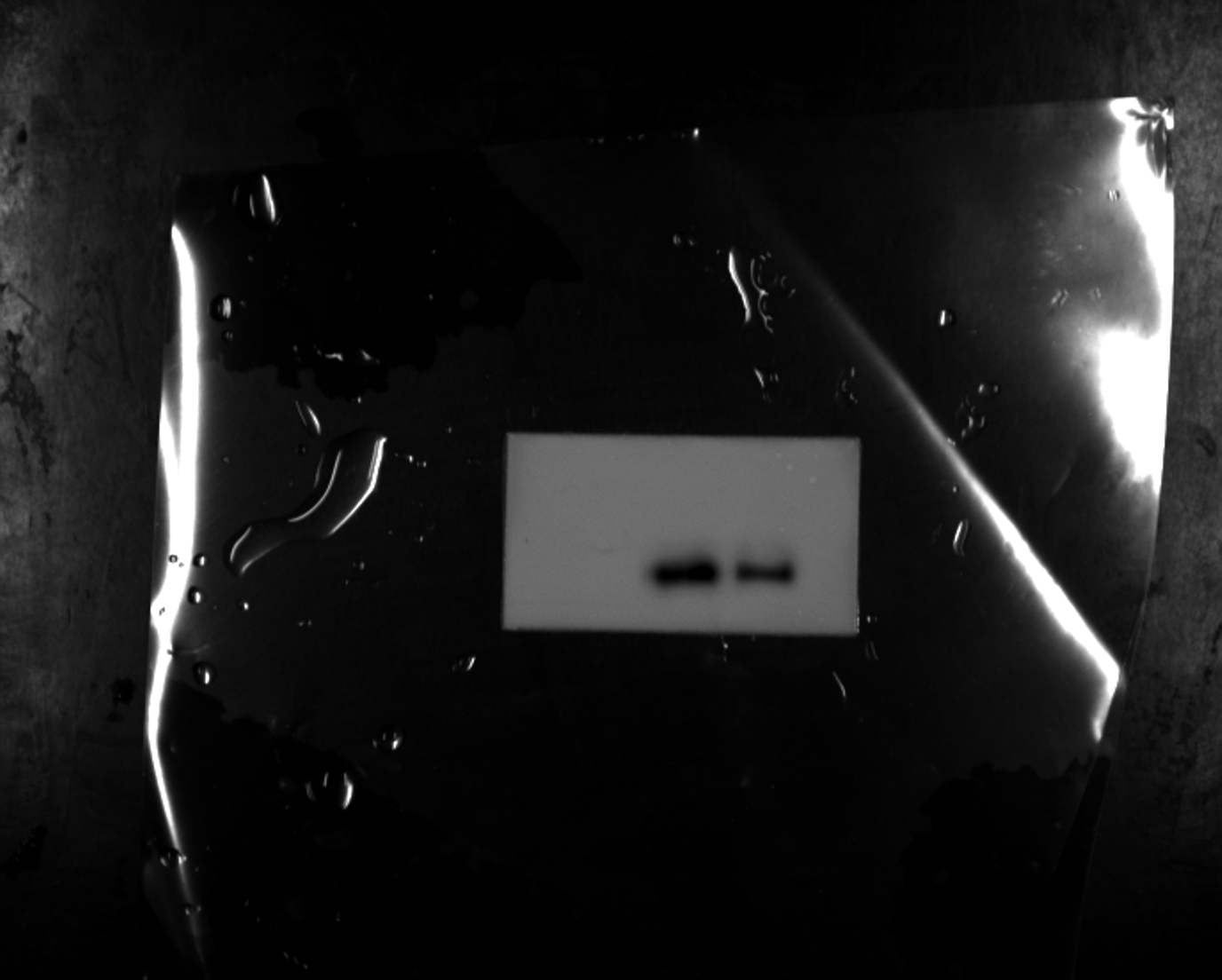

Supplement: Supplementary file 2 [file DataSheet_2.zip › Figure 8/Risk gene expression/ARX.Tif]

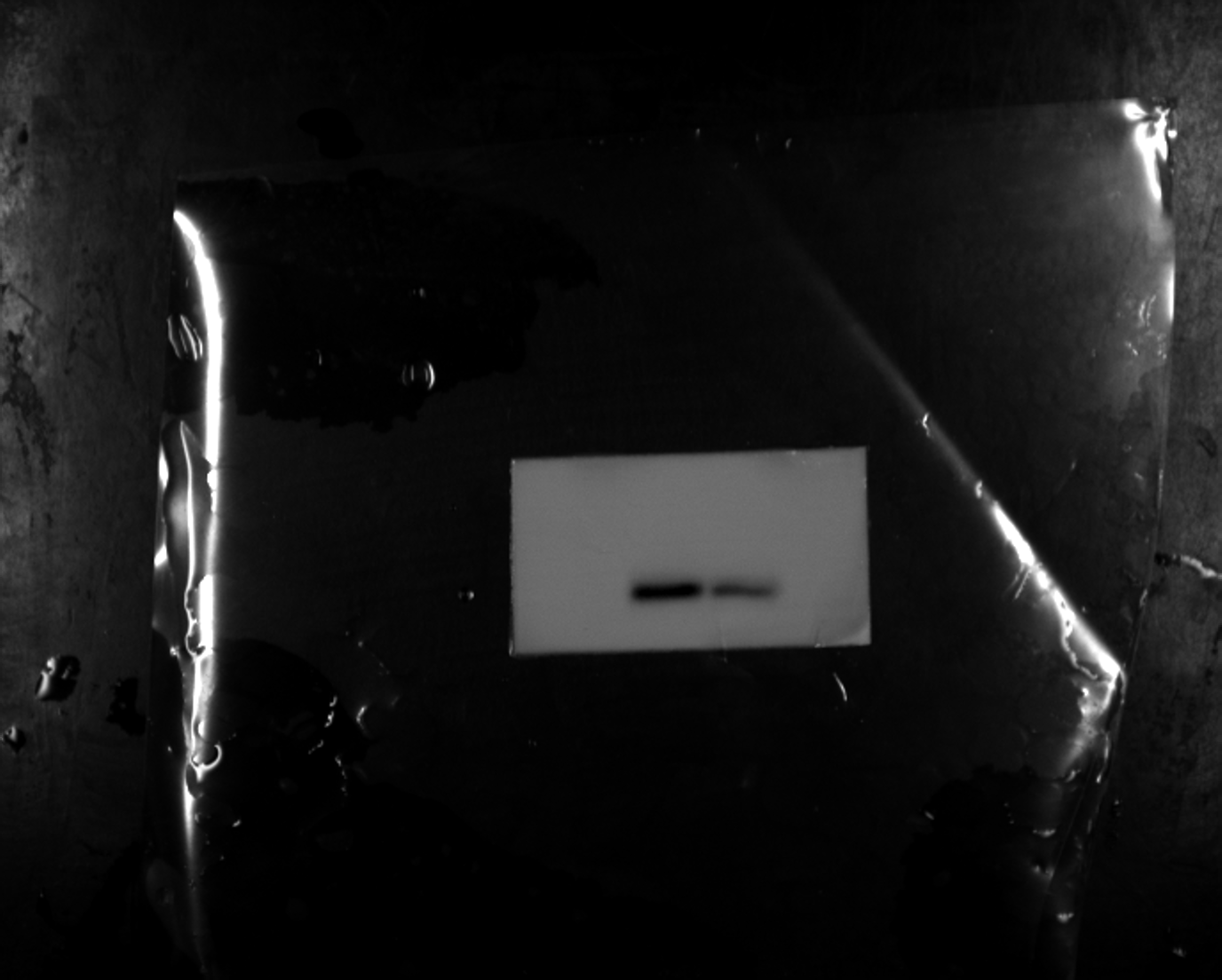

Supplement: Supplementary file 2 [file DataSheet_2.zip › Figure 8/Risk gene expression/CA12.Tif]

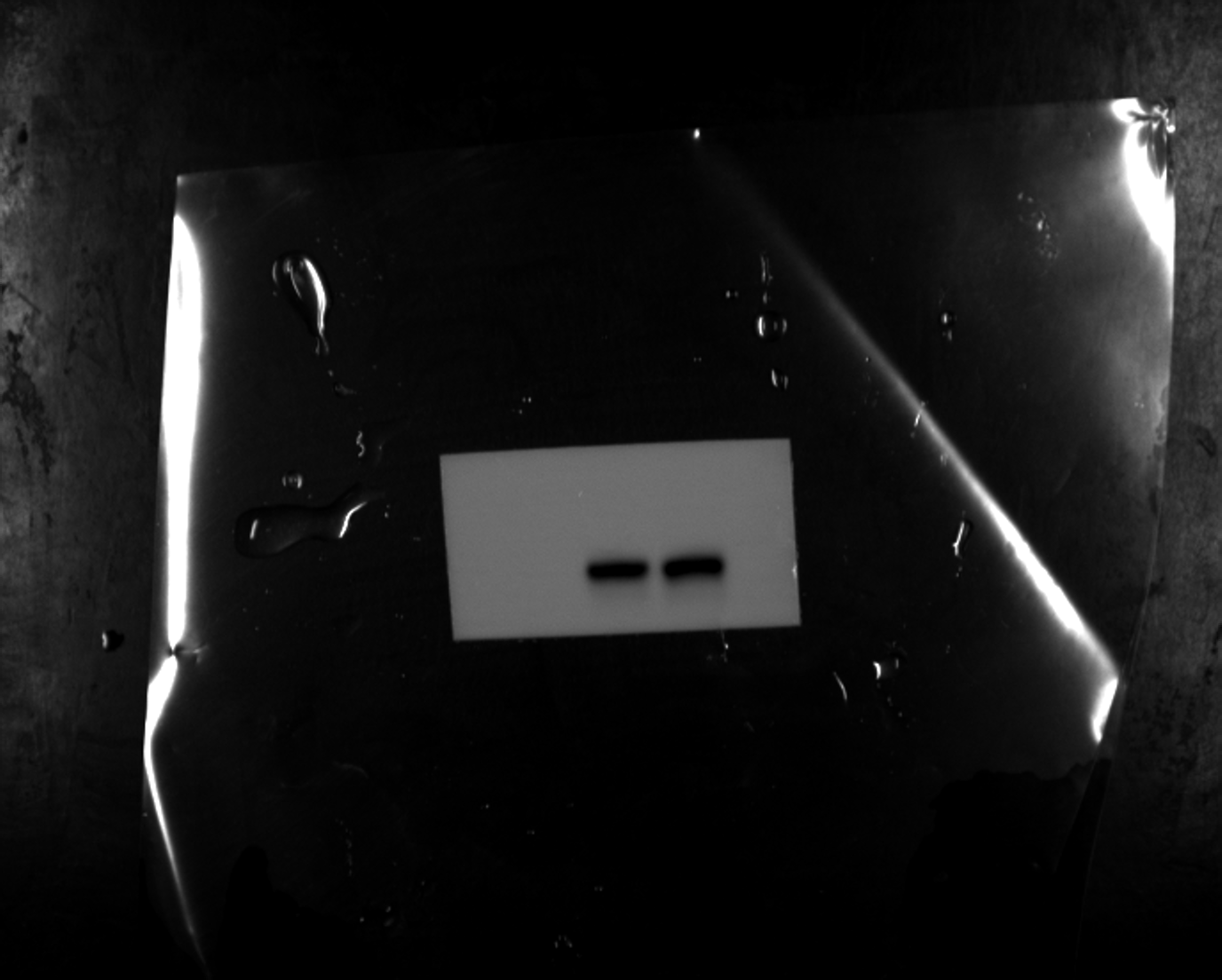

Supplement: Supplementary file 2 [file DataSheet_2.zip › Figure 8/Risk gene expression/GAPDH.Tif]

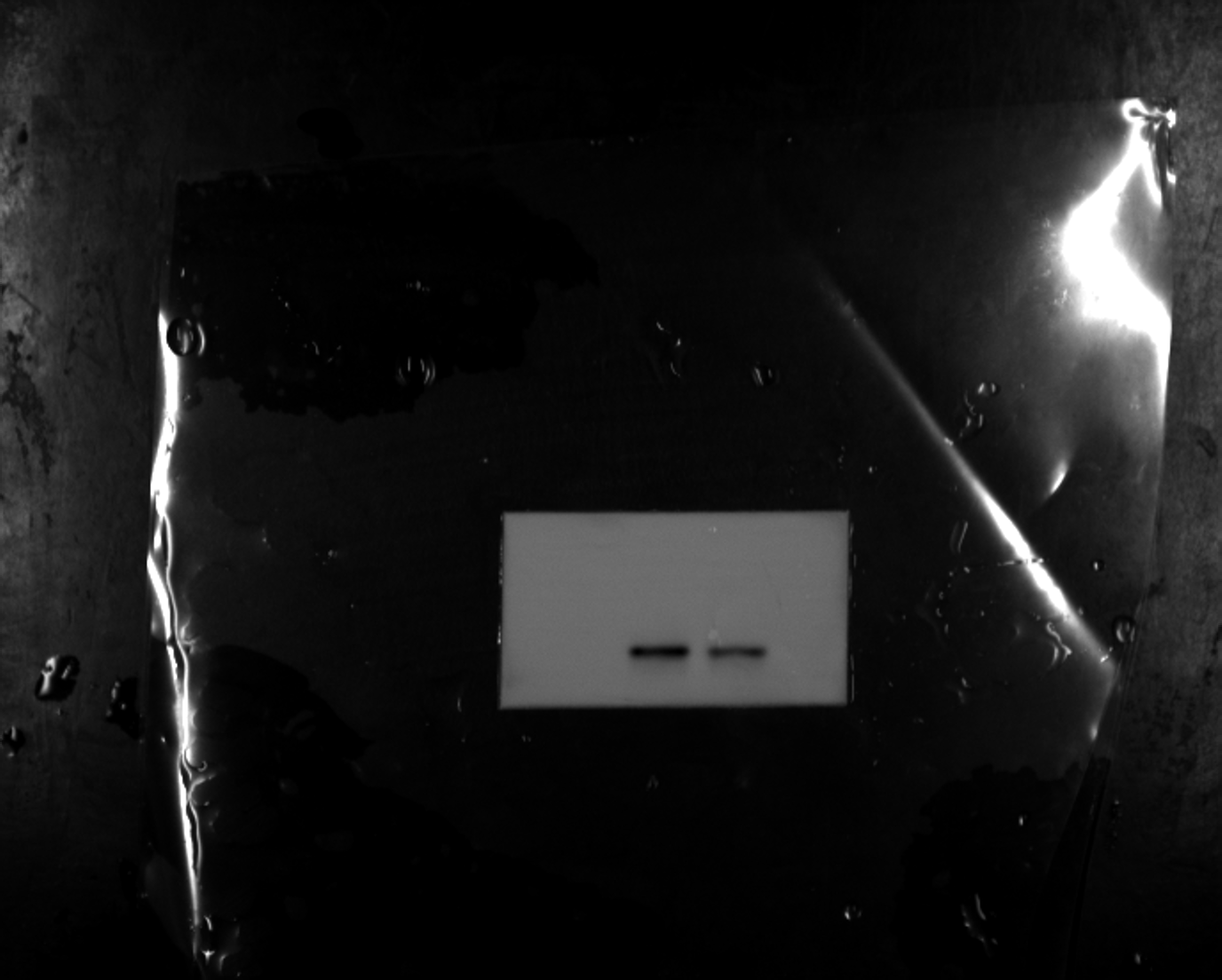

Supplement: Supplementary file 2 [file DataSheet_2.zip › Figure 8/Risk gene expression/MGLL.Tif]

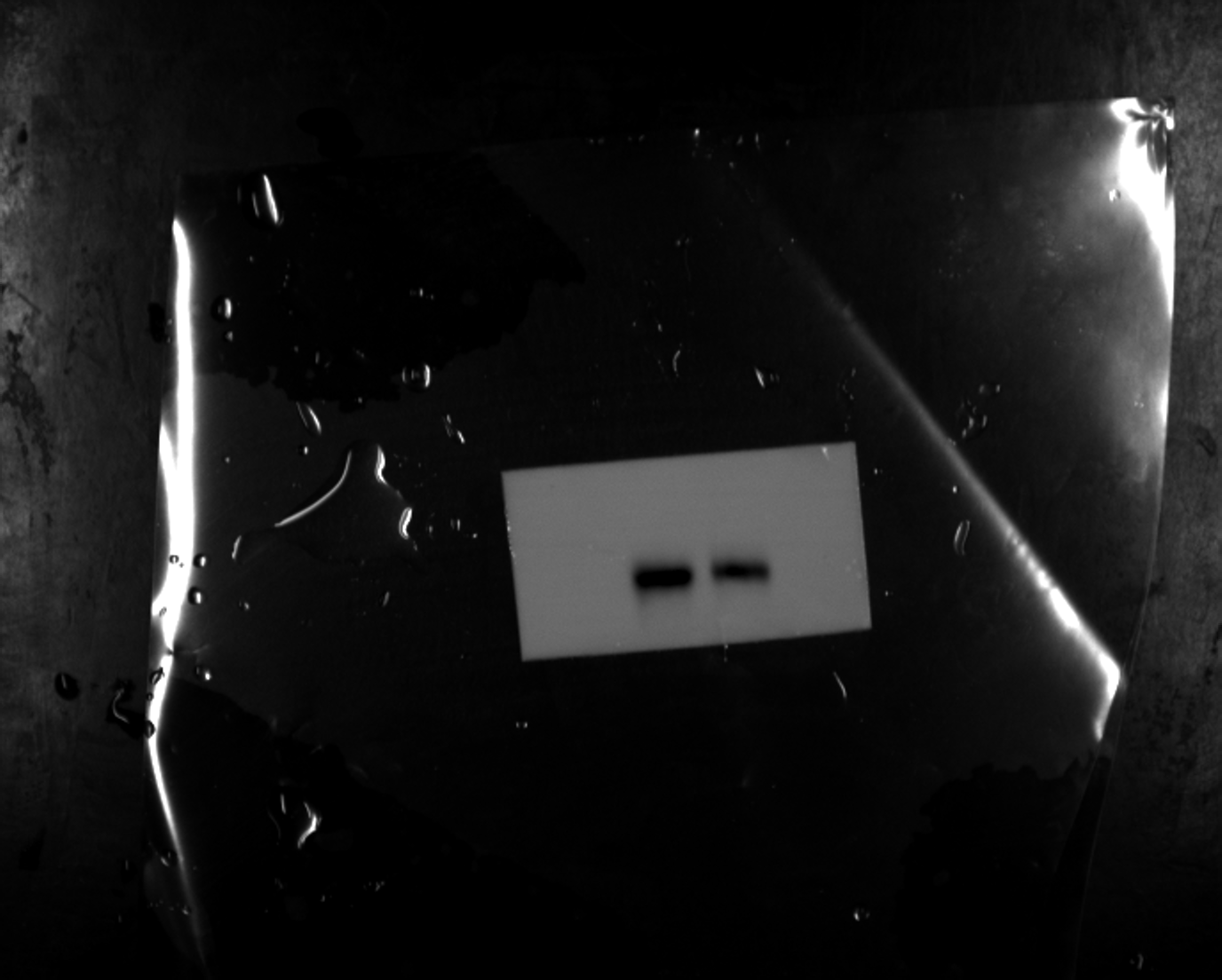

Supplement: Supplementary file 2 [file DataSheet_2.zip › Figure 8/Risk gene expression/MMP9.Tif]

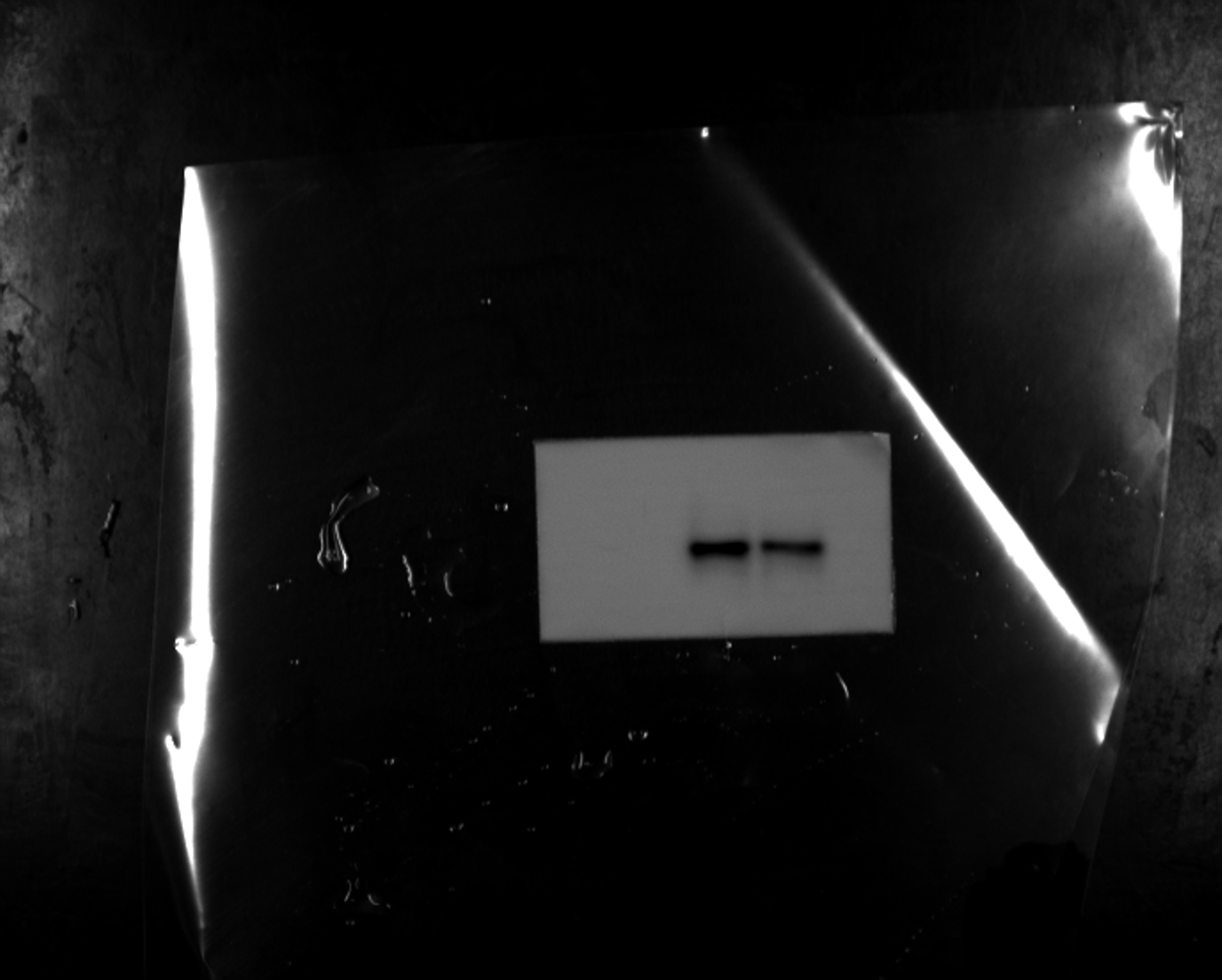

Supplement: Supplementary file 2 [file DataSheet_2.zip › Figure 8/Risk gene expression/S100A13.Tif]

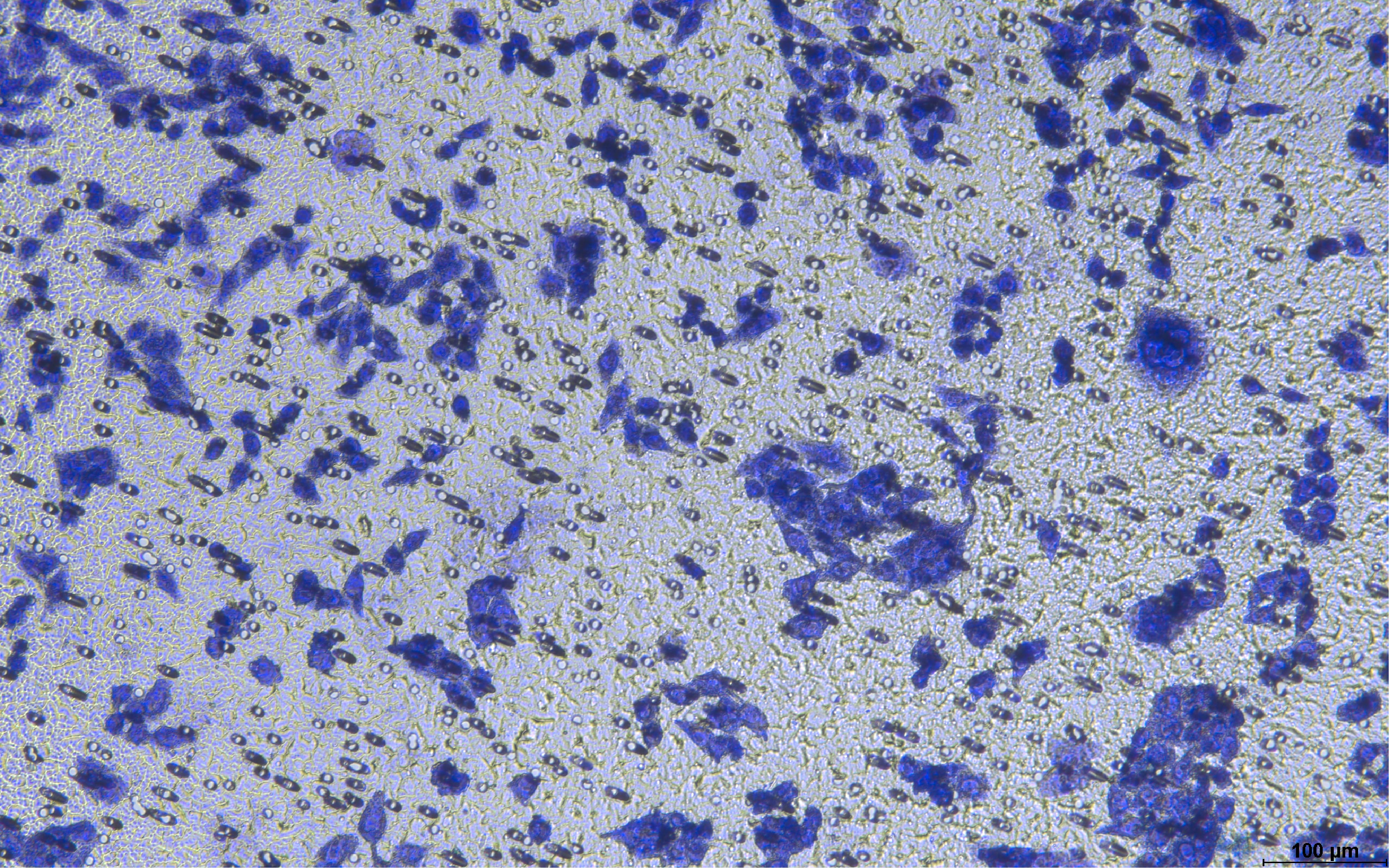

Supplement: Supplementary file 2 [file DataSheet_2.zip › Figure 8/Transwell images/Control.jpg]

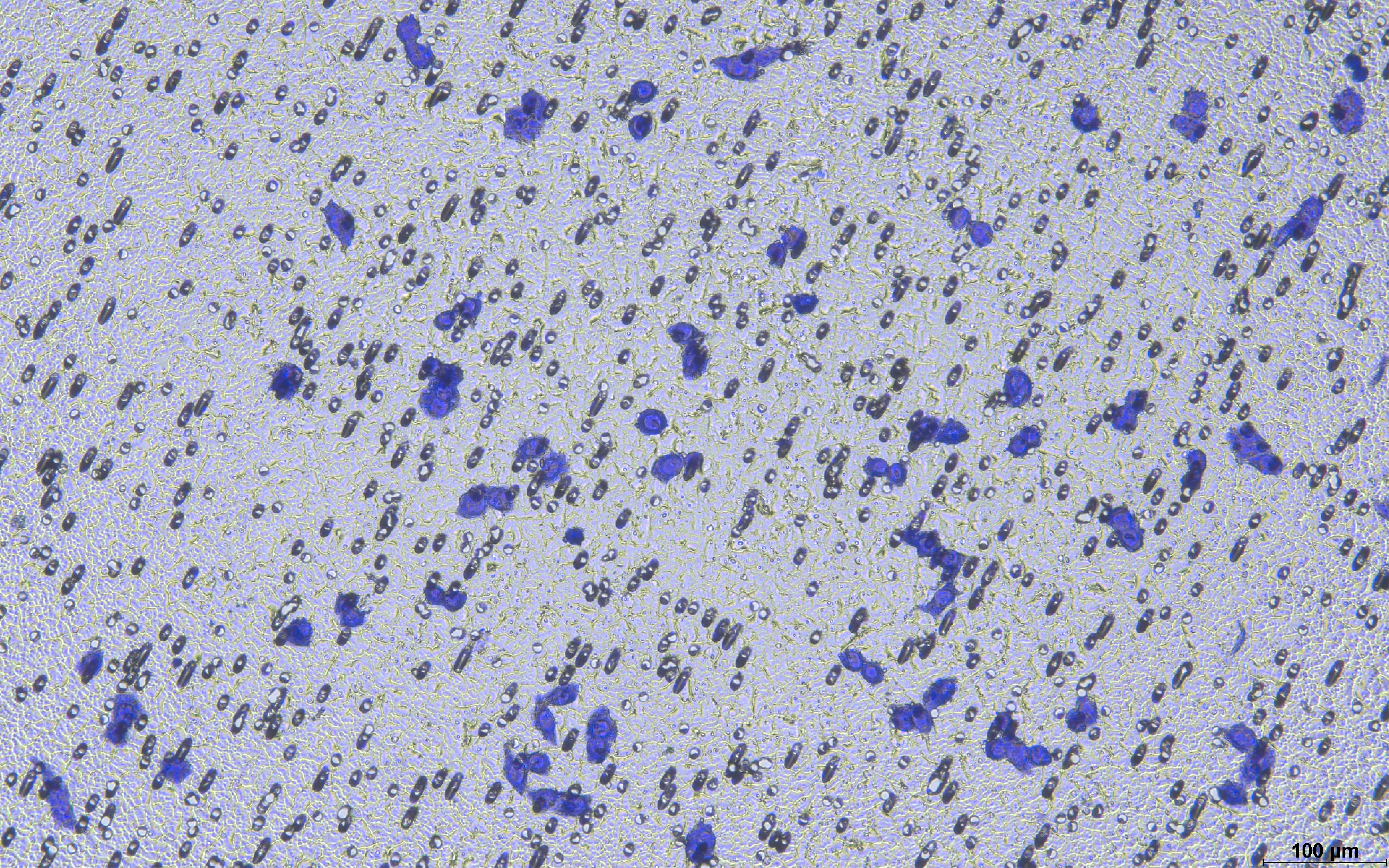

Supplement: Supplementary file 2 [file DataSheet_2.zip › Figure 8/Transwell images/siRNA1.jpg]

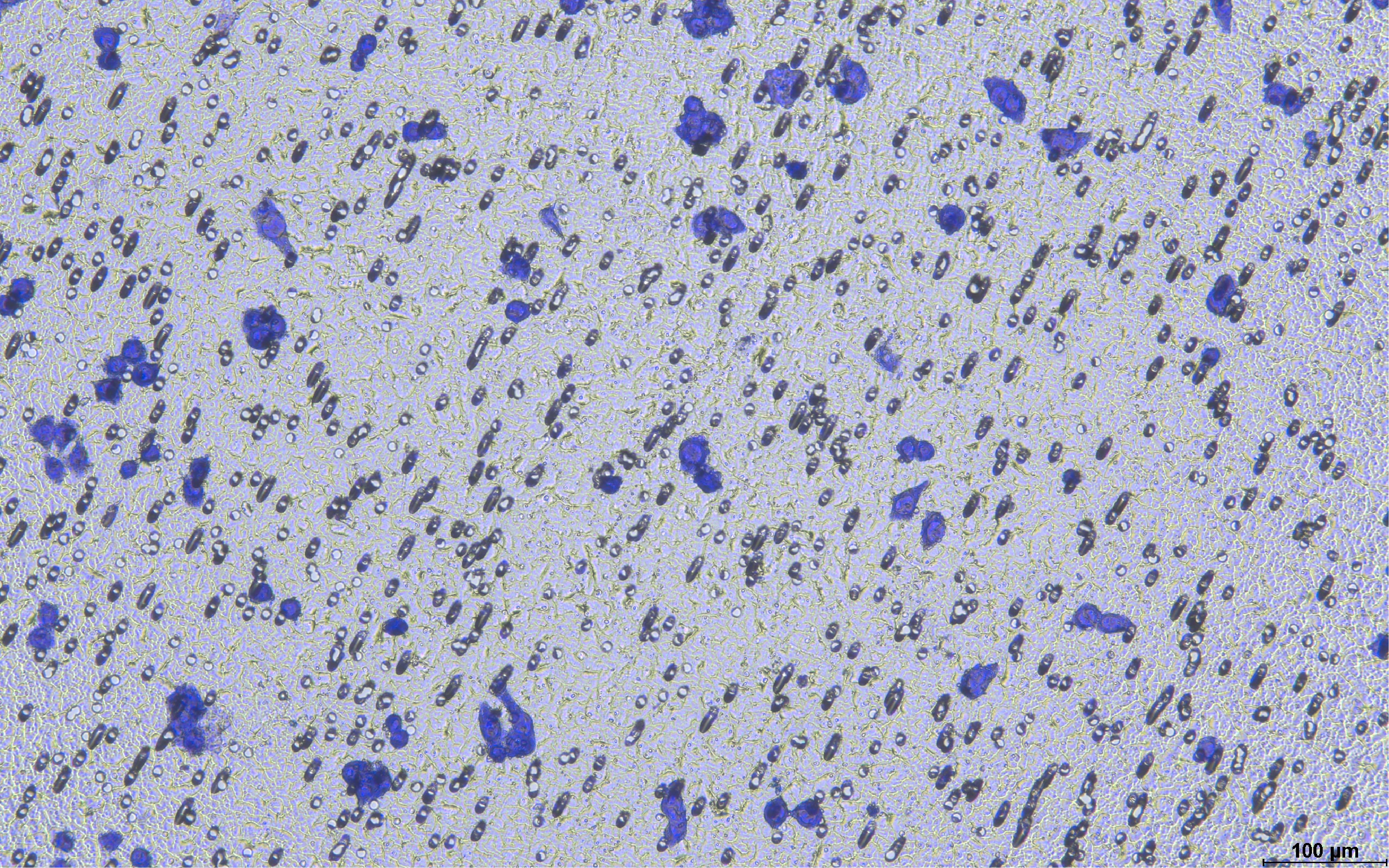

Supplement: Supplementary file 2 [file DataSheet_2.zip › Figure 8/Transwell images/siRNA2.jpg]

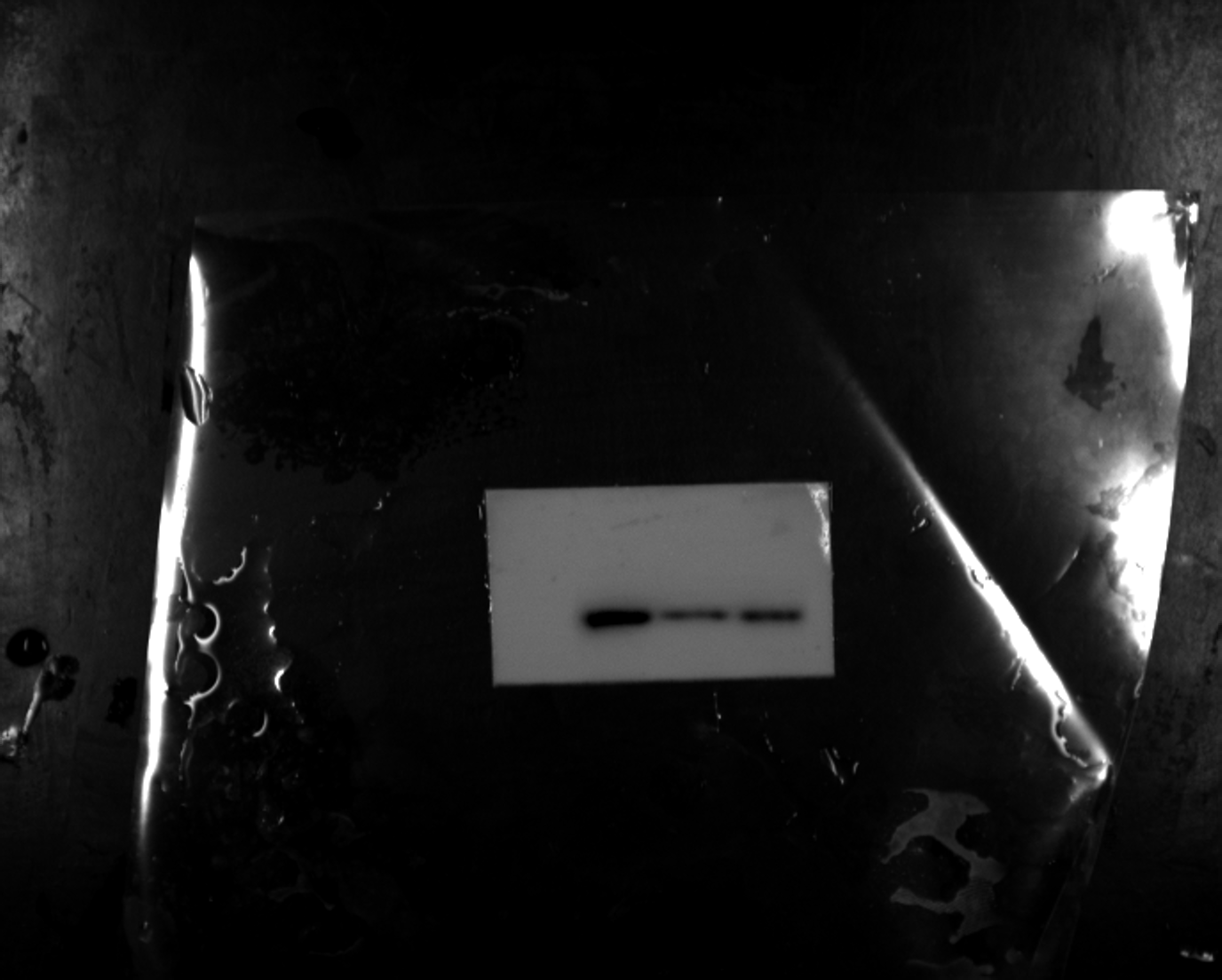

Supplement: Supplementary file 2 [file DataSheet_2.zip › Figure 9/CD276 expression/CA12.Tif]

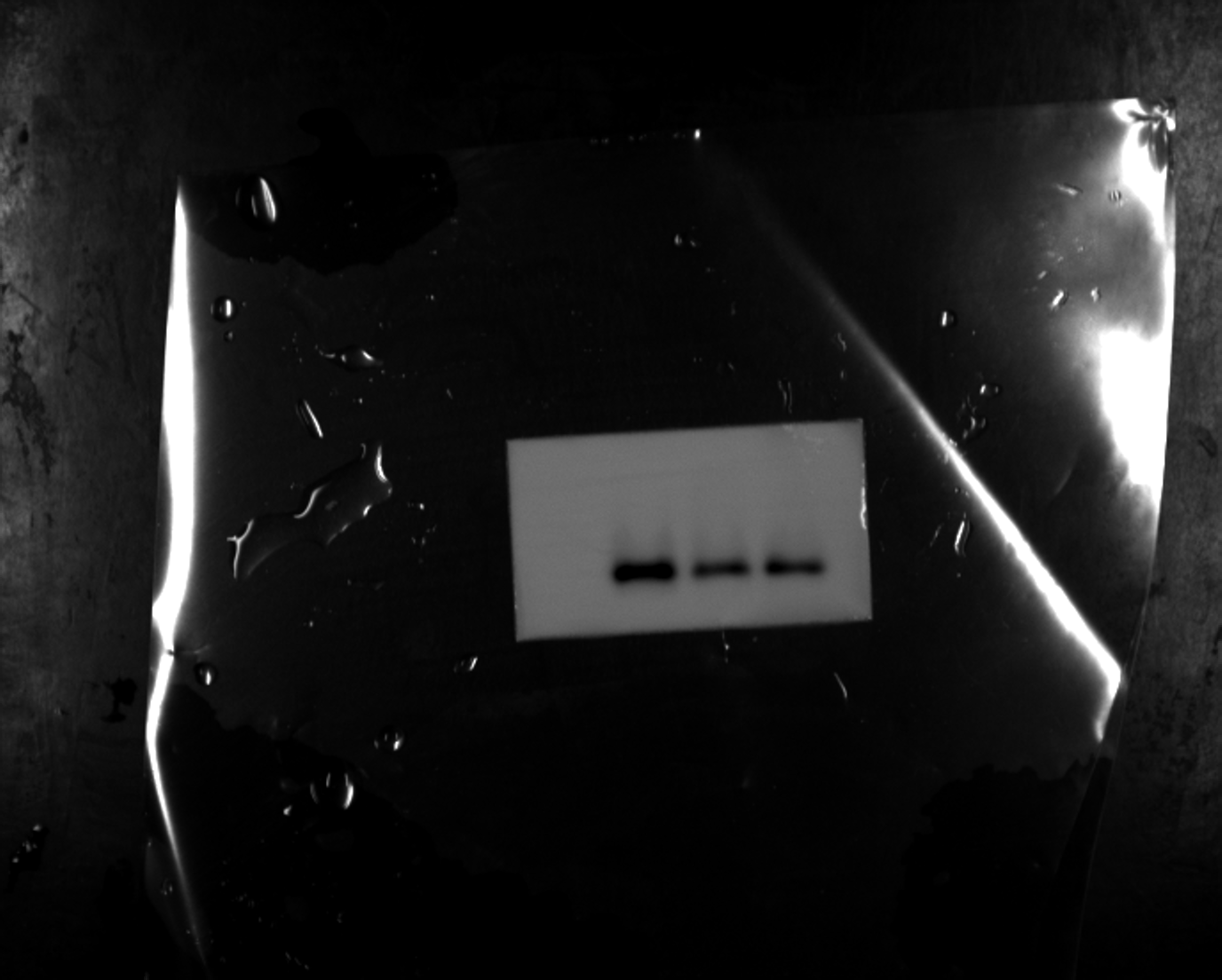

Supplement: Supplementary file 2 [file DataSheet_2.zip › Figure 9/CD276 expression/CD276.Tif]

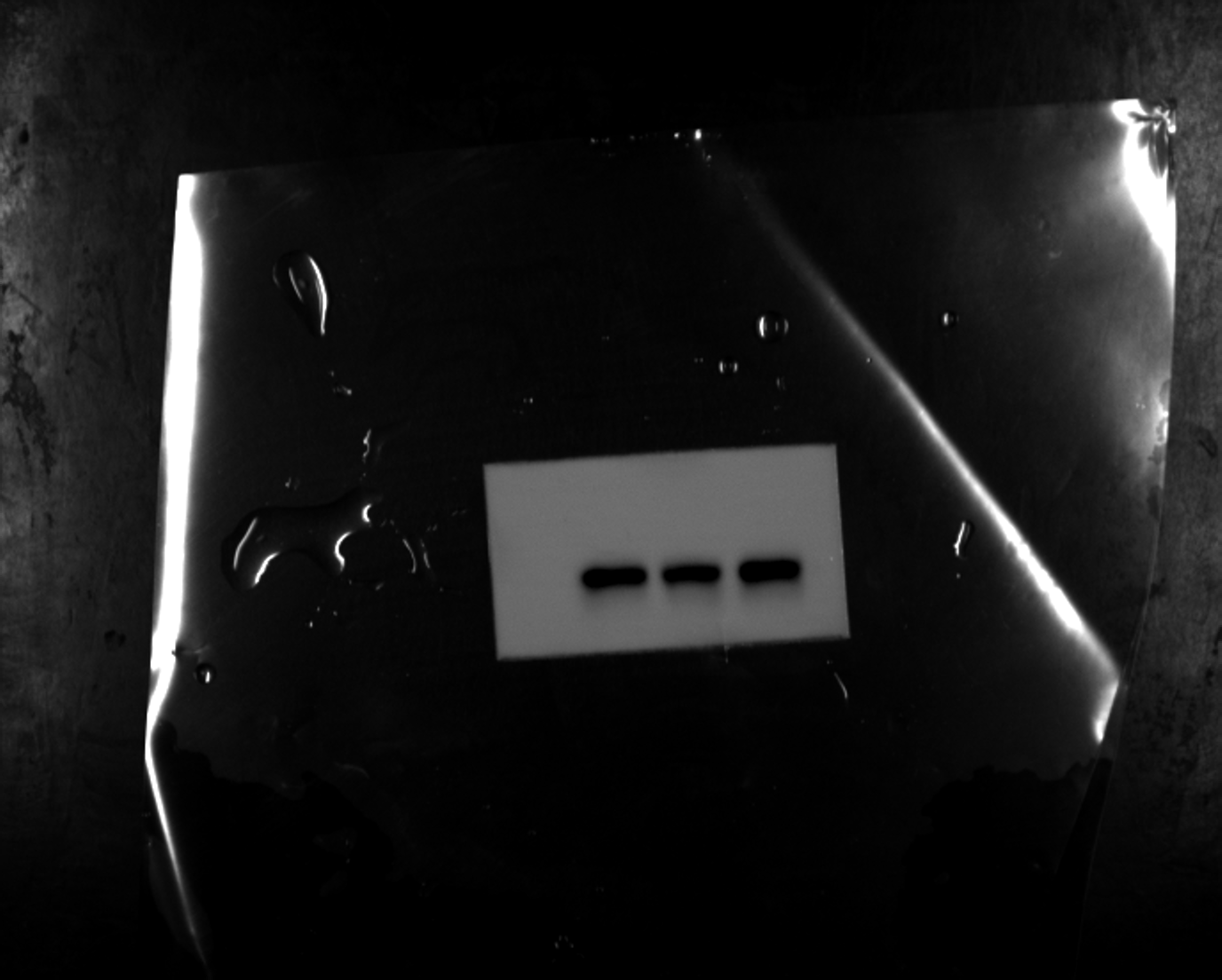

Supplement: Supplementary file 2 [file DataSheet_2.zip › Figure 9/CD276 expression/GAPDH.Tif]

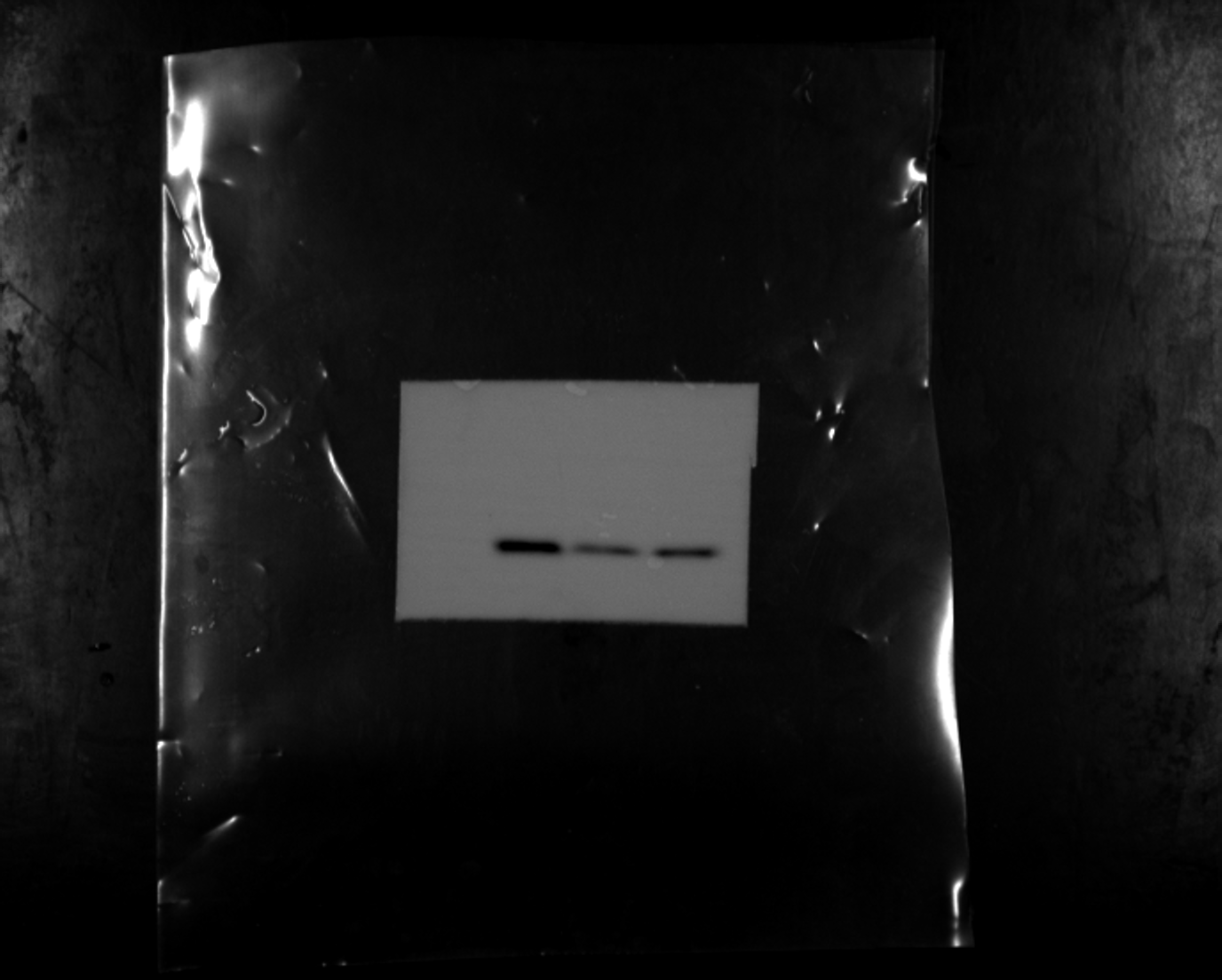

Supplement: Supplementary file 2 [file DataSheet_2.zip › Figure 9/Cell cycle protein western blot/CellCycle_CA12.Tif]

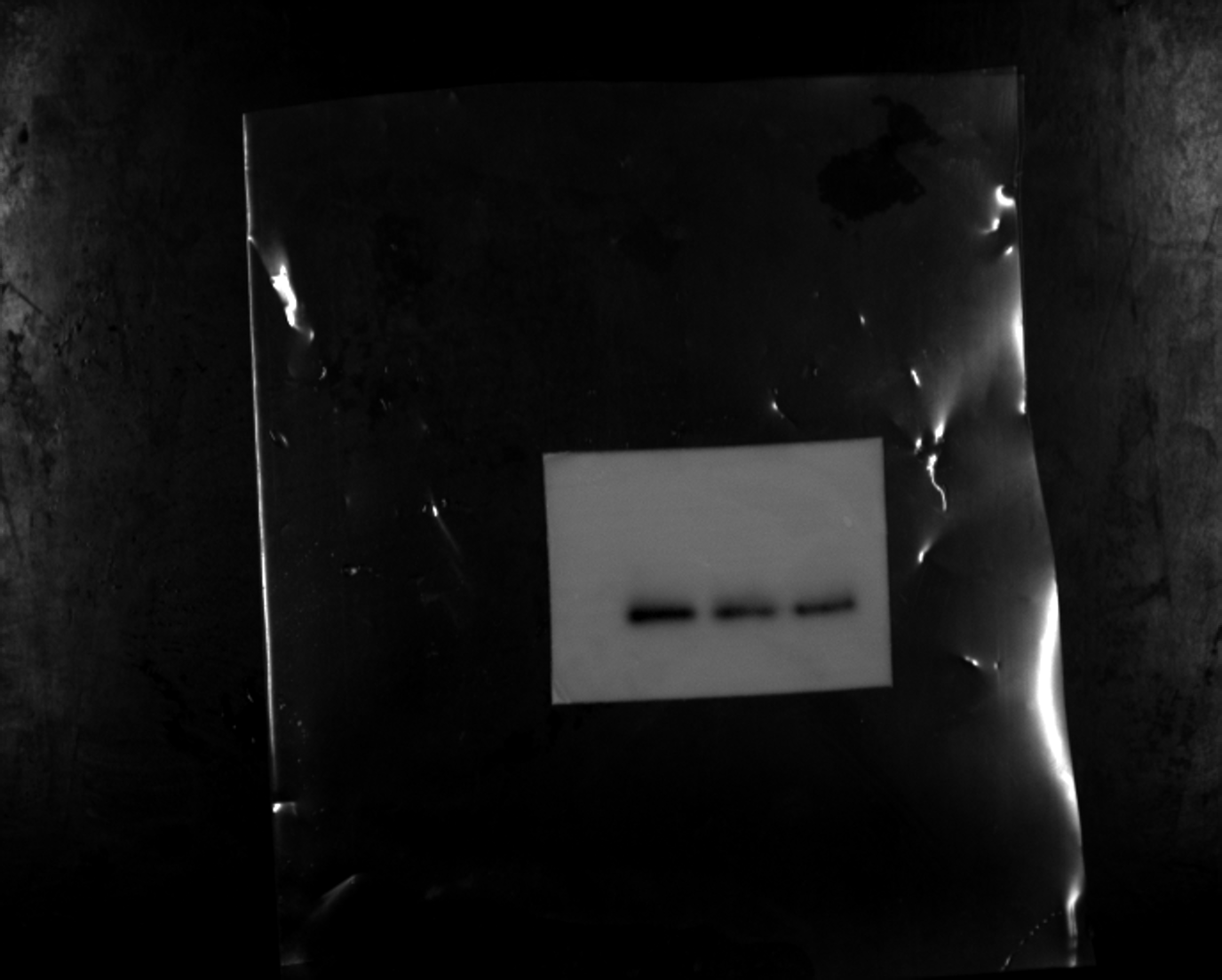

Supplement: Supplementary file 2 [file DataSheet_2.zip › Figure 9/Cell cycle protein western blot/CellCycle_CDK4.Tif]

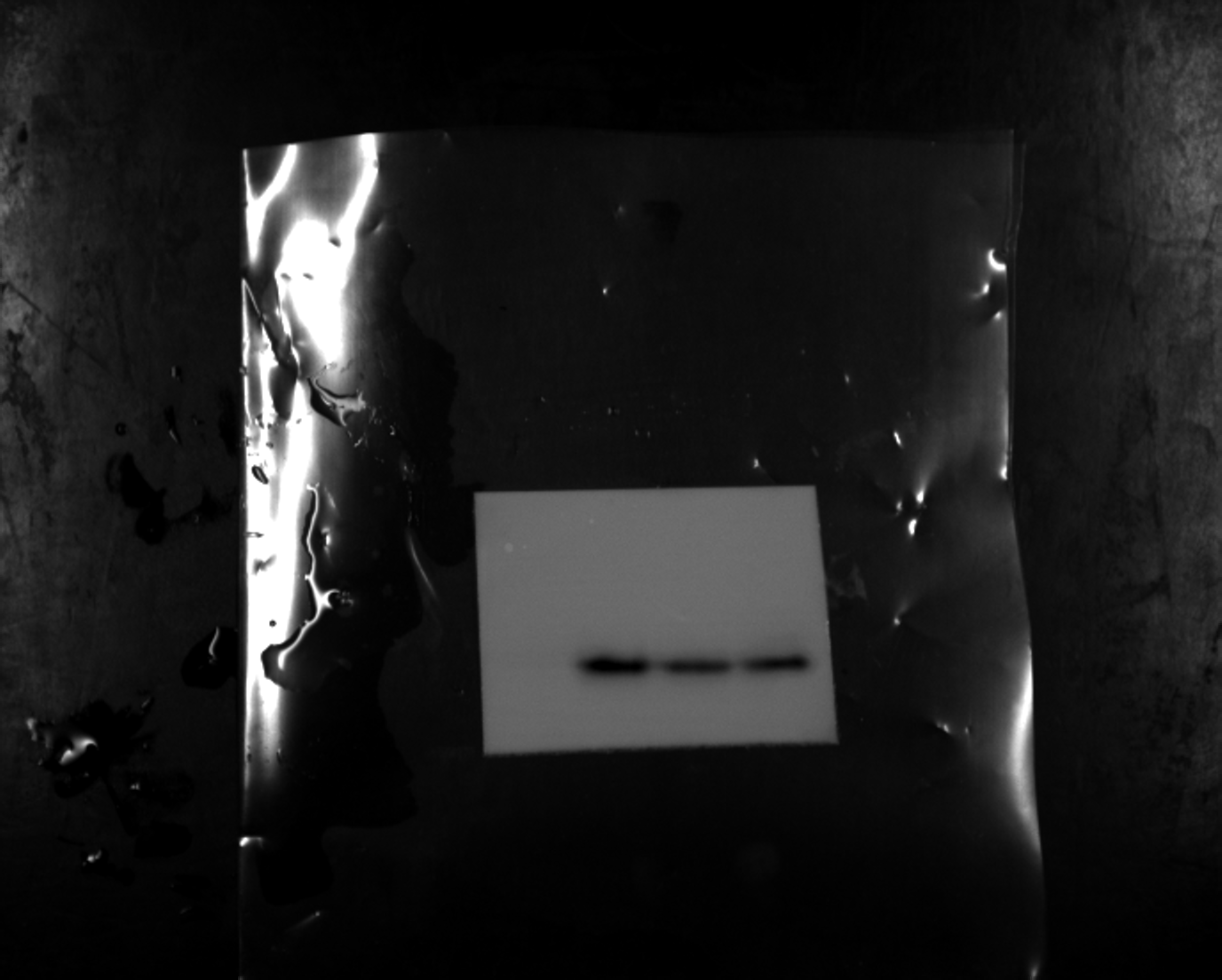

Supplement: Supplementary file 2 [file DataSheet_2.zip › Figure 9/Cell cycle protein western blot/CellCycle_CDK6.Tif]

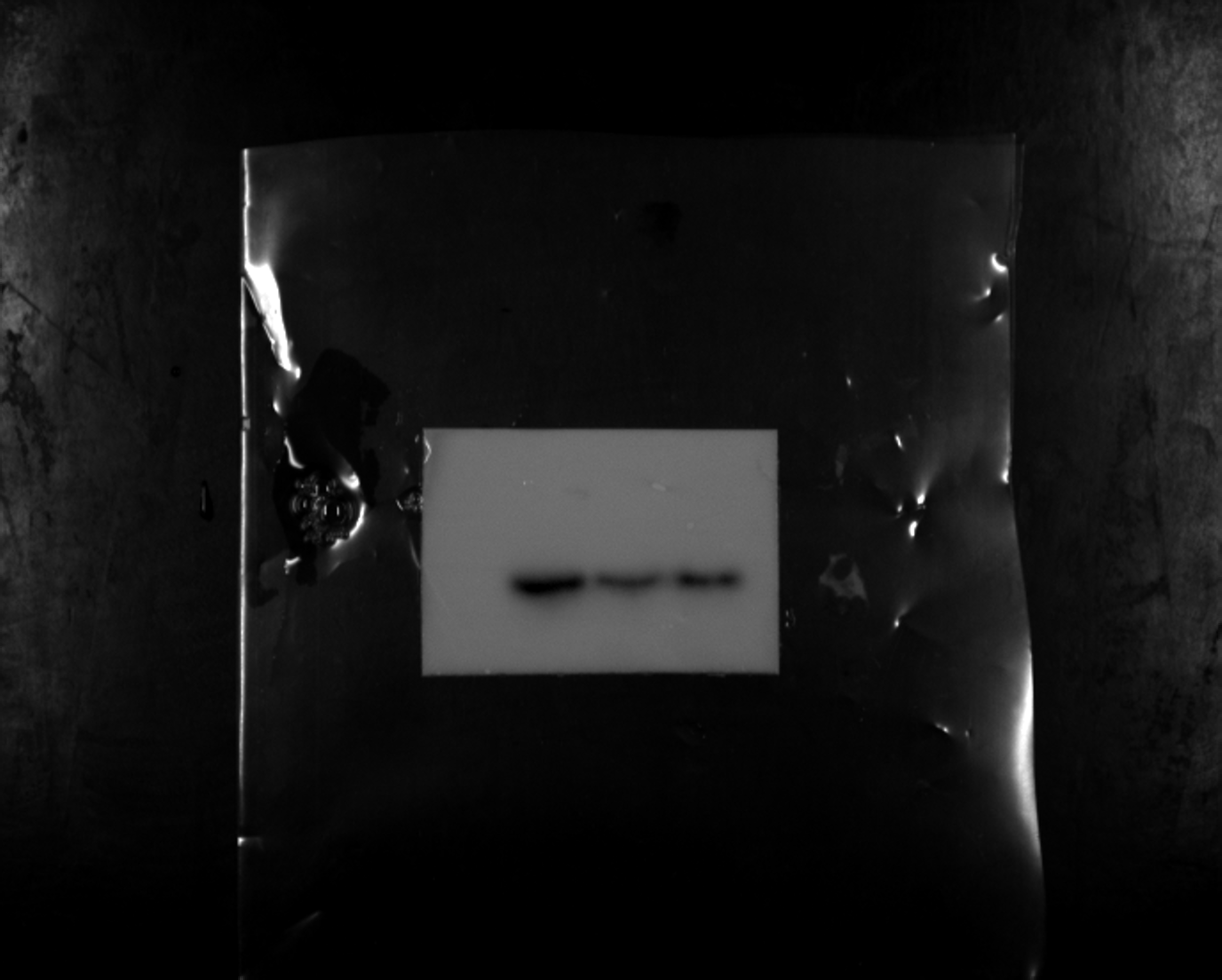

Supplement: Supplementary file 2 [file DataSheet_2.zip › Figure 9/Cell cycle protein western blot/CellCycle_CyclinD1.Tif]

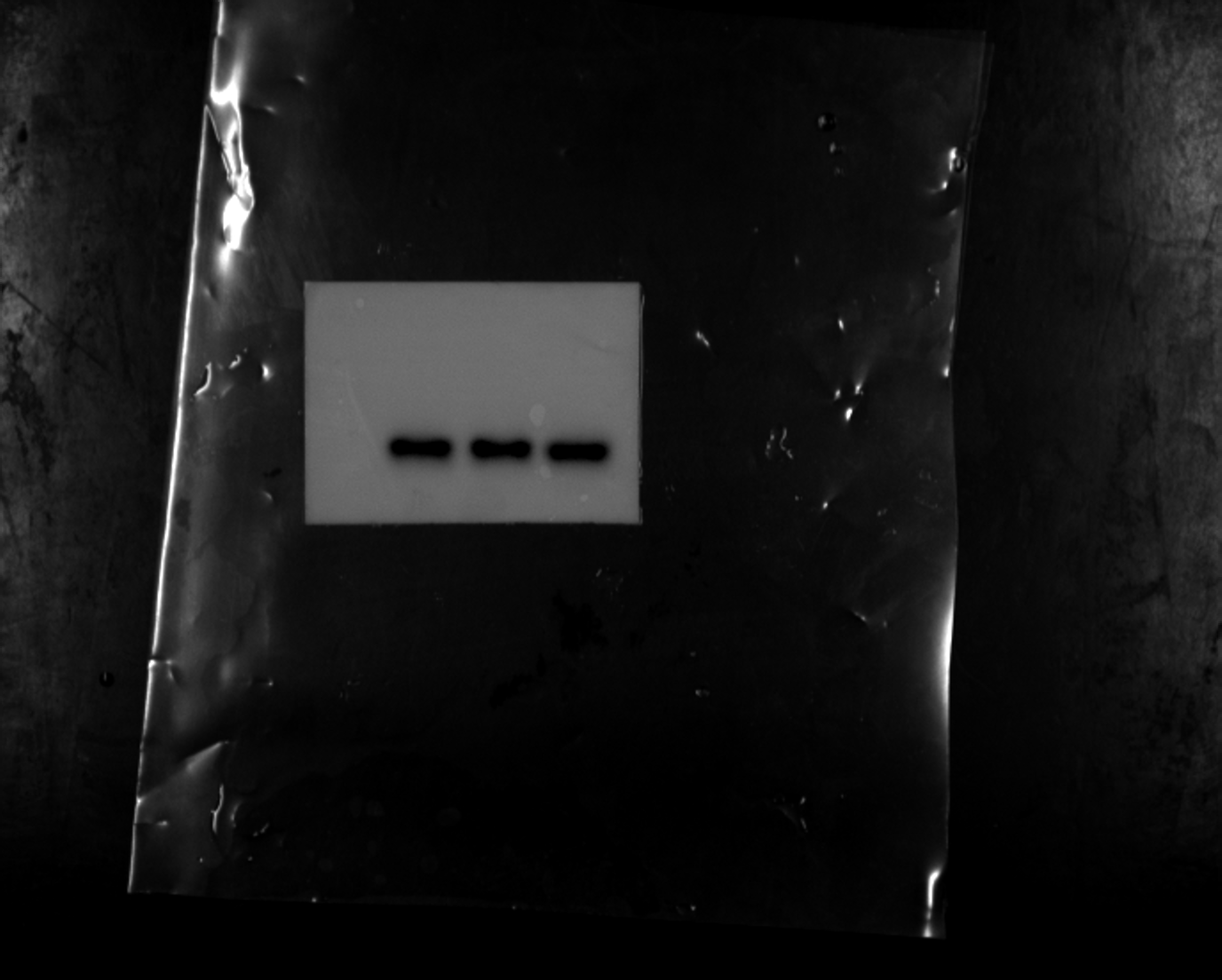

Supplement: Supplementary file 2 [file DataSheet_2.zip › Figure 9/Cell cycle protein western blot/CellCycle_GAPDH.Tif]

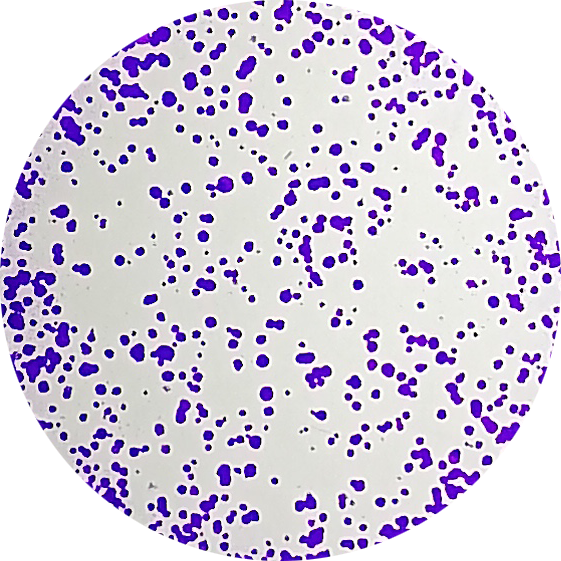

Supplement: Supplementary file 2 [file DataSheet_2.zip › Figure 9/Clone formation images/Control.png]

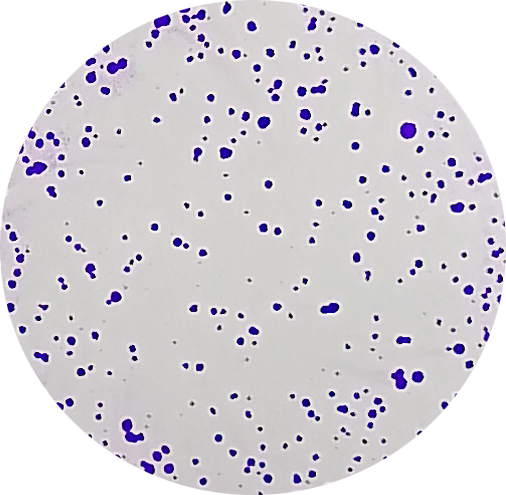

Supplement: Supplementary file 2 [file DataSheet_2.zip › Figure 9/Clone formation images/siRNA1.png]

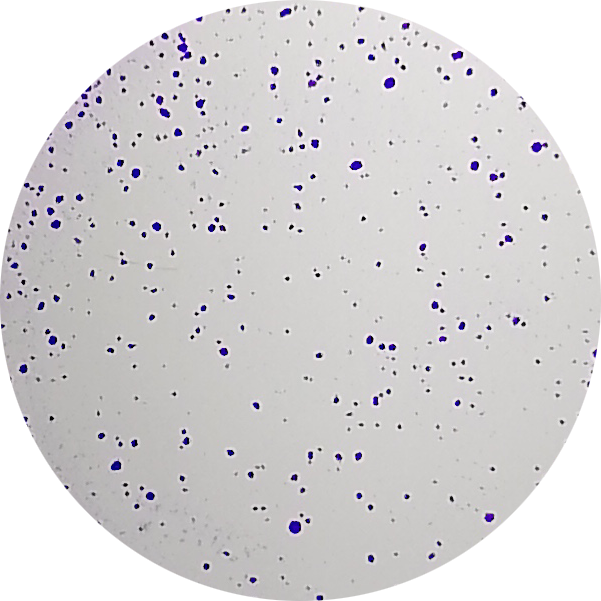

Supplement: Supplementary file 2 [file DataSheet_2.zip › Figure 9/Clone formation images/siRNA2.png]

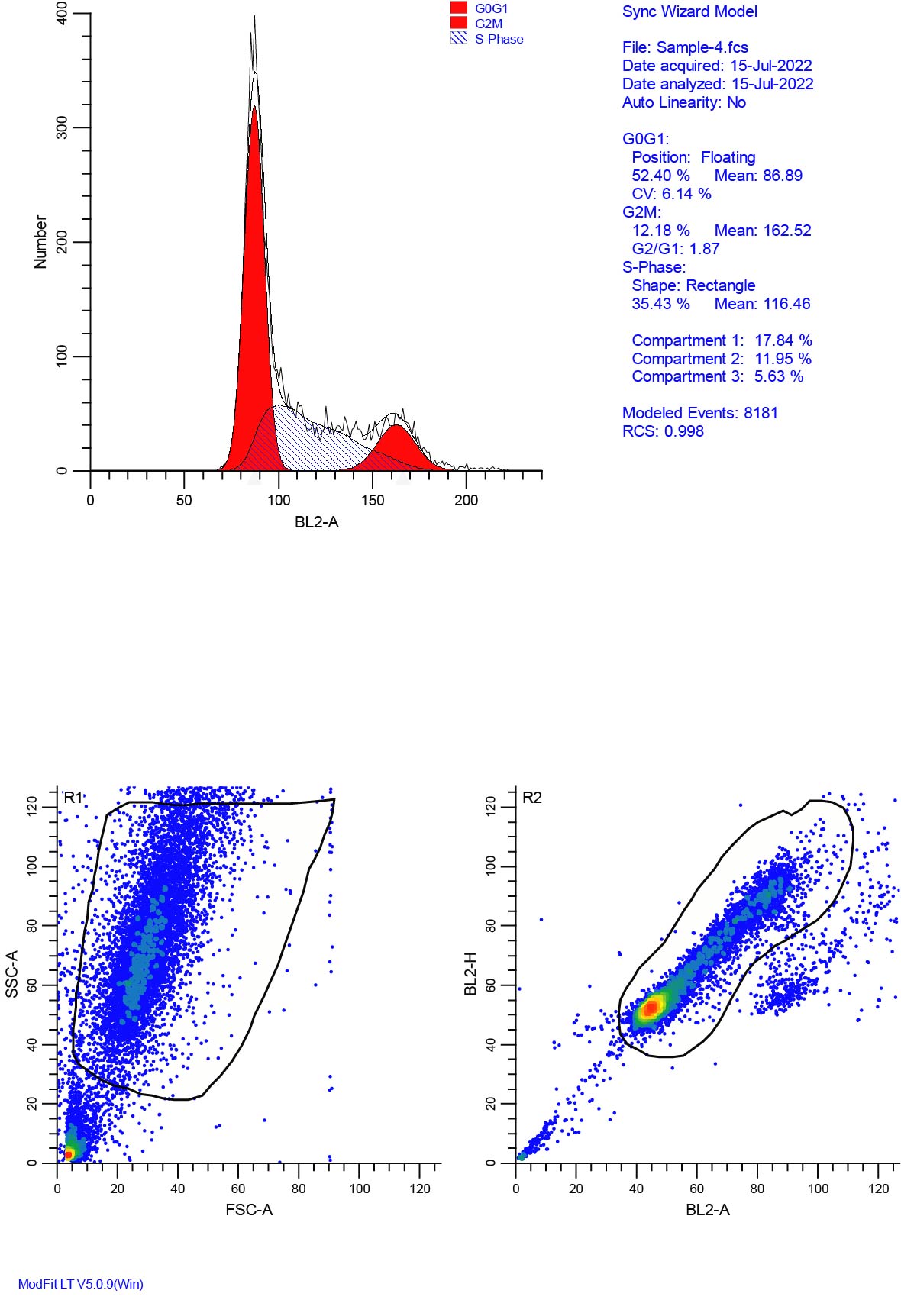

Supplement: Supplementary file 2 [file DataSheet_2.zip › Figure 9/Flow cytometry cell cycle/Control.jpg]

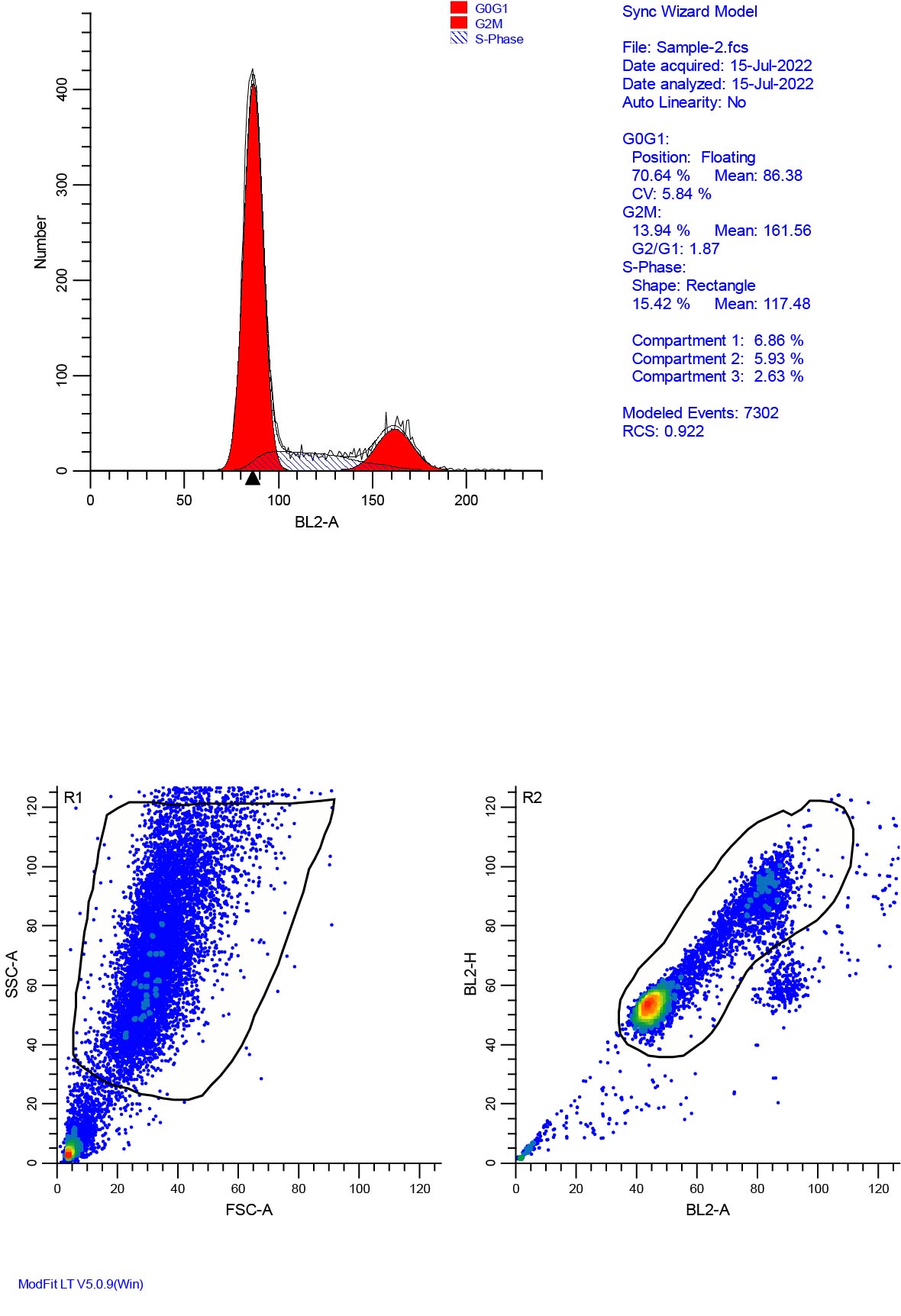

Supplement: Supplementary file 2 [file DataSheet_2.zip › Figure 9/Flow cytometry cell cycle/siRNA1.jpg]

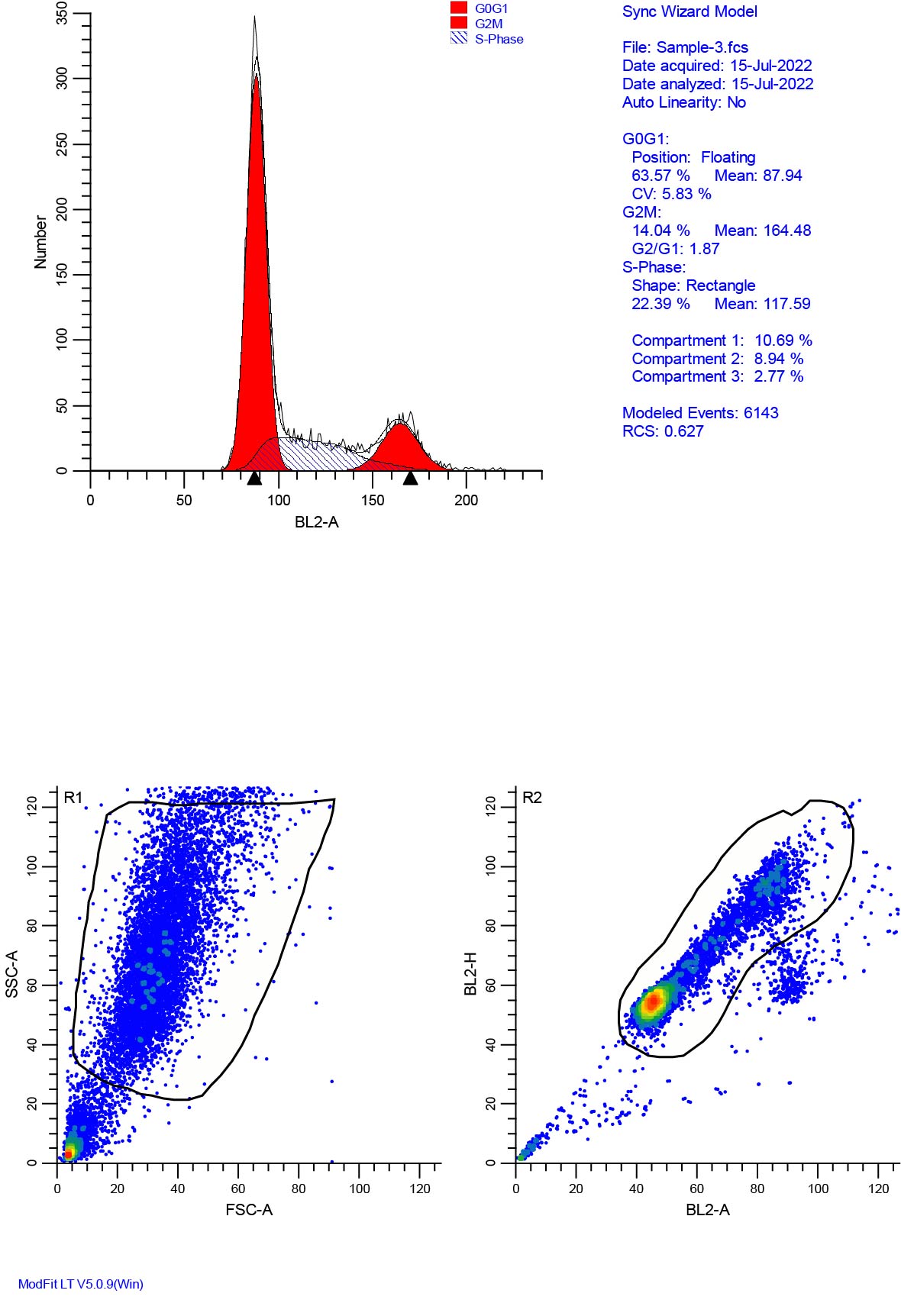

Supplement: Supplementary file 2 [file DataSheet_2.zip › Figure 9/Flow cytometry cell cycle/siRNA2.jpg]

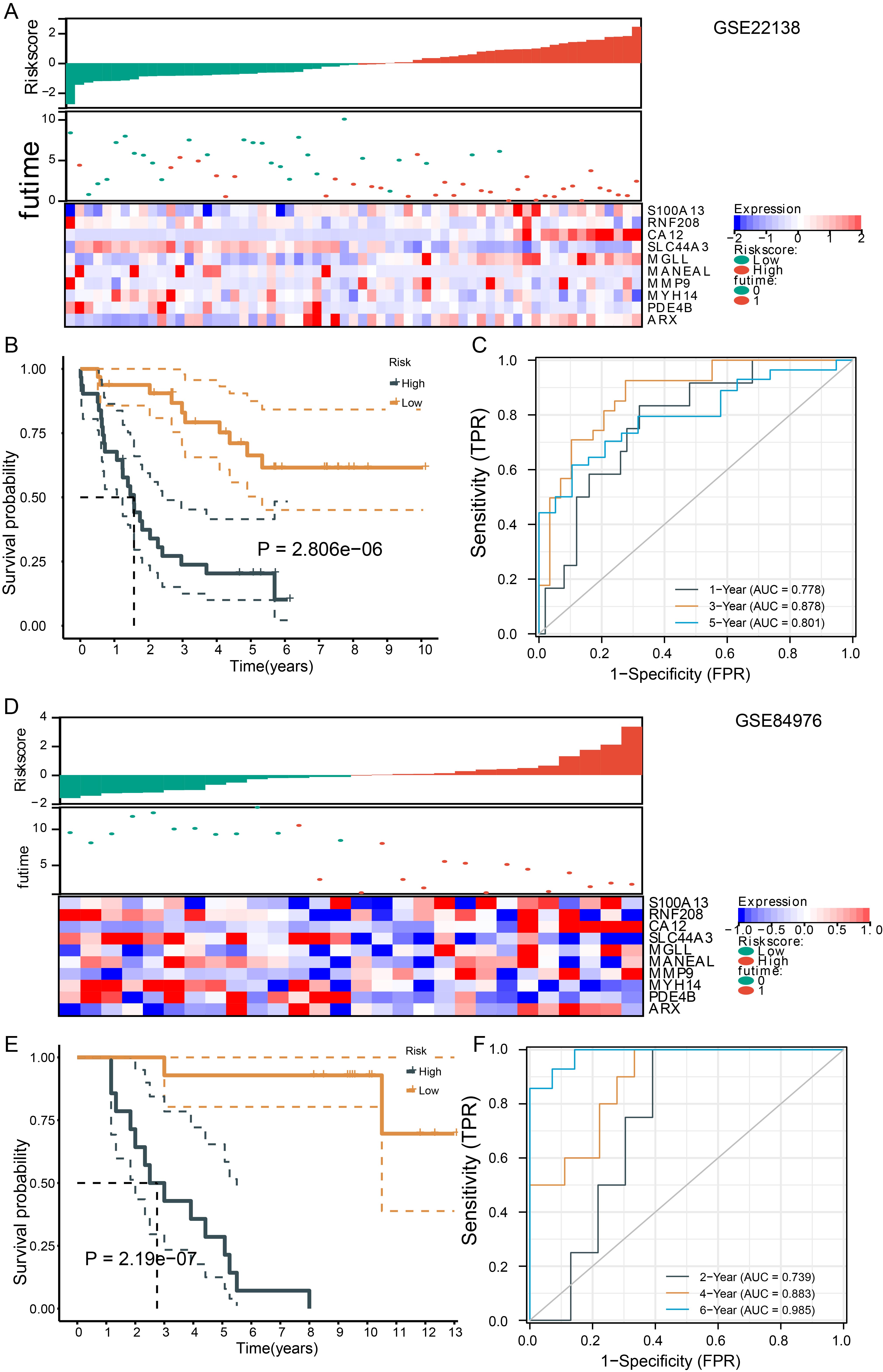

Supplement: Supplementary file 3 [file Image_1.jpeg]
