# Supplementary material for: The construction of a hypoxia-based signature identified CA12 as a risk gene affecting uveal melanoma cell malignant phenotypes and immune checkpoint expression
Source: Front Oncol. 2022 Sep 26;12:1008770. doi: 10.3389/fonc.2022.1008770 (PMC9548707; doi:10.3389/fonc.2022.1008770)
Supplement: Supplementary file 4 [file Table_1.docx]

**Supplementary Table S1** The antibody source used for western blot

| Antibody | Source |
| --- | --- |
| CA12 | Cell signaling technology, cat.#5864 |
| ARX | Cell signaling technology, cat.#8688 |
| MGLL | Cell signaling technology, cat.#14197 |
| MMP9 | Cell signaling technology, cat.#13667 |
| S100A13 | Proteintech, 14987-1-AP |
| HIF-1α | Cell signaling technology, cat.#36169 |
| GAPDH | Proteintech, 60004-1-Ig |
| E-cadherin | Cell signaling technology, cat.#14472 |
| N-cadherin | Cell signaling technology, cat.#13116 |
| Vimentin | Cell signaling technology, cat.#5741 |
| CyclinD1 | Cell signaling technology, cat.#55506 |
| CDK4 | Cell signaling technology, cat.#12790 |
| CDK6 | Cell signaling technology, cat.#13331 |
| CD276 | Cell signaling technology, cat.#14058 |
